# Comparison of leaf transcriptome in response to *Rhizoctonia solani* infection between resistant and susceptible rice cultivars

**DOI:** 10.1186/s12864-020-6645-6

**Published:** 2020-03-19

**Authors:** Wei Shi, Shao-Lu Zhao, Kai Liu, Yi-Biao Sun, Zheng-Bin Ni, Gui-Yun Zhang, Hong-Sheng Tang, Jing-Wen Zhu, Bai-Jie Wan, Hong-Qin Sun, Jin-Ying Dai, Ming-Fa Sun, Guo-Hong Yan, Ai-Min Wang, Guo-Yong Zhu

**Affiliations:** Jiangsu Coastal Area Institute of Agricultural Sciences, Yancheng City, Jiangsu Province, 224002 P. R. China

**Keywords:** Rice, Sheath blight, Transcriptome, RNA-seq, Molecular breeding

## Abstract

**Background:**

Sheath blight (SB), caused by *Rhizoctonia solani*, is a common rice disease worldwide. Currently, rice cultivars with robust resistance to *R. solani* are still lacking. To provide theoretic basis for molecular breeding of *R. solani*-resistant rice cultivars, the changes of transcriptome profiles in response to *R. solani* infection were compared between a moderate resistant cultivar (Yanhui-888, YH) and a susceptible cultivar (Jingang-30, JG).

**Results:**

In the present study, 3085 differentially express genes (DEGs) were detected between the infected leaves and the control in JG, with 2853 DEGs in YH. A total of 4091 unigenes were significantly upregulated in YH than in JG before infection, while 3192 were significantly upregulated after infection. Further analysis revealed that YH and JG showed similar molecular responses to *R. solani* infection, but the responses were earlier in JG than in YH. Expression levels of trans-cinnamate 4-monooxygenase (C4H), ethylene-insensitive protein 2 (EIN2), transcriptome factor WRKY33 and the KEGG pathway plant-pathogen interaction were significantly affected by *R. solani* infection. More importantly, these components were all over-represented in YH cultivar than in JG cultivar before and/or after infection.

**Conclusions:**

These genes possibly contribute to the higher resistance of YH to *R. solani* than JG and were potential target genes to molecularly breed *R. solani*-resistant rice cultivar.

## Background

To prevent pathogen invasion, plants have evolved innate immune system, which can effectively detect extracellular and intracellular signals of pathogens and then activate physiological and biochemical responses to resist pathogens, such as enhancing the hormone defense pathway, switching off plant growth and regulating the expressions of immunity-related genes [1]. Based on these features, scientists can breed pathogen-resistant cultivars for agricultural production [2].

Sheath blight (SB) caused by *Rhizoctonia solani* is one of the three major diseases in rice. The pathogen has an extremely broad range of hosts and can infect more than 32 families and 188 genera of plant species [3]. *R. solani* can be characterized into different sub-groups known as anastomosis groups (AGs). Among them, rice is specifically infected by *R. solani* Kuhn AG1-1A [4]. To breed SB-resistant rice cultivar, large-scale screening has been performed on various cultivated germplasms and wild species. However, only a few varieties showed partial resistance to SB [5], which may hinder the development of SB-resistant rice cultivars [2]. Molecular breeding is an effective method for fast screening of cultivars with specific traits. To facilitate the molecular breeding of SB-resistant rice, knowledges in relation to innate immune responses to SB infection are required.

Traditional genetic analysis revealed that SB resistance in rice was a typical quantitative trait controlled by multiple genes [6]. Up to date, approximately 50 SB-resistant quantitative trait loci (SBR QTLs) have been detected on all 12 chromosomes in rice [7, 8]. However, most of them did not show consistent and stable resistance to SB, which might be affected by environmental parameters [9]. Thus, no effective QTLs have been obtained for molecular breeding of SB-resistant rice cultivar. High-throughput screening of more SB-resistant QTLs is still required. Using Robust-Long-serial analysis of gene expression technique (RL-SAGE) and microarrays, Venu et al. [10] investigated mRNA changes of rice after infection, identifying some resistance-related genes. Similarly, Yuan et al. [11] compared transcriptome changes of *R. solani*-resistant and susceptible rice cultivars in response to *R. solani* using microarrays and the results suggested that receptor-like kinases and jasmonic acid signaling pathway might play important roles in host resistance to *R. solani*. Compared with the microarray method, RNA-sequencing (RNA-Seq) provides much more detailed information on specific transcript expression patterns [12]. Moreover, RNA-seq shows higher accuracy and sensitivity than microarrays or other traditional methods to explore differentially expressed genes, discovery of novel transcripts and detection of gene expression [13, 14]. With help of RNA-seq, Xia et al. [15] has investigated transcriptome changes of *R. solani* AG1IA isolated from rice, soybean and corn, providing new insights into mechanisms underlying host preference and pathogenesis. Based on transcriptome analyses of *R. solani*, Rao et al. [16] found polygalacturonase (PG) determined infection virulence of *R. solani*, and transgenic rice cultivar stably expressing RNA interference (RNAi) targeting on PG showed resistance to sheath blight. These results provided new information of the pathogenic process. Zhang et al. [17, 18] compared the transcriptome changes of leaves between TeQing (a moderately resistant cultivar) and Lemont (a susceptible cultivar) cultivars in response to *R. solani* infection. The results showed that regulation of photosynthesis, photorespiration, jasmonic acid and phenylpropanoid pathways might contribute to rice resistance to *R. solani*. However, the main difference between the resistant and susceptible rice cultivars was the timing of responses after infection [17]. The resistance of rice plants to *R. solani* was affected by environmental parameters [9]. Moreover, *R. solani* mutations could overcome rice resistance introduced by single resistant genes [19]. Breeding of rice cultivars with stable SB-resistance requests deep understanding of molecular mechanisms, which must base on broad exploration of innate immune genes in rice. The current knowledges in this area are still not robust enough. Investigations on more rice cultivars are still necessary to collect information of general resistant genes.

Yanhui-888 (YH) is a new two-line restorer cultivar bred by the Jiangsu Coastal Area Institute of Agricultural Sciences (Yancheng, China). As officially assessed by the Jiangsu Academy of Agricultural Sciences (Nanjing, China), Yanhui-888 displays moderate resistance to *R. solani* [20]. The rice cultivar Jingang-30 (JG) is susceptible to various infections, including *R. solani*. In the present study, these two varieties were infected with *R. solani* and then RNA-seq was applied to explore transcriptional responses in rice leaves. These results would provide a comprehensive view of the transcriptome regulation after *R. solani* infection in rice plants. The identified candidate genes might be used for molecular breeding of SB-resistant rice cultivars in future.

## Methods

### Sample collection and *R. solani* inoculation

Seeds of Yanhui-888 (YH) and Jingang-30 (JG) were provided by the Jiangsu Coastal Agricultural Research Institute and the Jiangsu Academy of Agricultural Sciences (Nanjing, China), respectively. The seeds were sterilized in 4% sodium hypochlorite (NaClO) for 10 min, rinsed with distilled water for three times and then immersed in distilled water for 2 days. Afterwards, germinated seeds were moved into plastic plots (10 cm × 10 cm × 10 cm) containing sterile nutrient soils. Rice seedlings were cultured in a greenhouse at 25 ± 1 °C. The light cycle was 16 h: 8 h (light: dark) with the light intensity of approximately 13,200 lx. 1/2 Hoagland’s solution was used to irrigate rice seedlings daily. After 40 days, the seedlings at the middle tillering stage were used for inoculation.

*R. solani* strain RH-2 was kindly gifted by Jiangsu Academy of Agricultural Sciences and grew on potato dextrose agar (PDA) plates containing 50 μg/mL ampicillin. The inoculation was performed according to Xue et al. [21]. Wooden tips (1 cm long and 0.5 mm diameter) were sterilized at 121 °C for 20 min, placed on agar plates with *R. solani* and then cultured for 3 days. When these tips were covered with *R. solani*, they were inserted slightly into the second sheath of rice seedlings. Sterile tips without inoculum were used as the control. For each treatment, 10 plants were included. Afterwards, the culture temperature was adjusted to 28 °C and the humidity was adjusted to 100% RH. After 3 days, obvious symptoms of SB were observed. The parts of leaves displaying SB symptoms were collected. Samples from three plants were mixed as one and then stored at − 80 °C for RNA-seq. Three biological replicates were included for each treatment independently. In total, 12 samples were sequenced, including 2 varieties × 2 treatments (infected and uninfected) × 3 replicates. Infected samples were labeled as YH-1 and JG-1 and uninfected samples were labeled as YH-0 and JG-0.

### RNA extraction and sequencing

The total RNA was extracted using Trizol reagent (Invitrogen, USA) according to the manufacturer’s instructions. RNA concentration and quality were determined using NanoDrop 2000 spectrophotometer (Thermo, USA) and Agilent Bioanalyzer 2100 system (Agilent Technologies, CA, USA), respectively. Samples with RNA integrity number (RIN) higher than 8.0 were considered qualified.

mRNA was enriched using NEBNext Poly(A) mRNA Magnetic Isolation Module (NEB, USA). Sequencing libraries were constructed following the protocols of NEBNext Ultra directional RNA library prep kit for Illumina (NEB, USA). RNA molecules were fragmented using divalent cations with increasing temperature. The first strand cDNA was prepared using random hexamer primers and M-MuLV reverse transcriptase. The second strand cDNA was synthesized using DNA Polymerase I. Residual RNA was eliminated using RNase H and remaining overhangs were removed by exonuclease/polymerase activities. Afterwards, 3′ ends of DNA were adenylated, which were further ligated to NEBNext adaptor containing hairpin loop structure for hybridization. DNA fragments were cleaned up using AMPure XP system (Beckman Coulter, Beverly, USA). Next, samples were treated with 3 μl of USER enzyme (NEB, USA) at 37 °C for 15 min and the reaction was stopped by heating at 95 °C for 5 min. After amplification using Phusion High-Fidelity DNA polymerase, universal PCR primers and index (X) primers, and purification using AMPure XP system, the quality of library was monitored using the Agilent Bioanalyzer 2100 system. The concentrations of libraries were determined by real-time quantitative PCR (RT-qPCR). RNA-seq libraries were clustered on a cBot cluster generation system using an Illumina HiSeq 4000 PE cluster kit and finally sequenced on an Illumina Hiseq 2500 platform.

### Differentially expressed genes and qPCR validation

Adaptors, low quality reads (with > 50% bases having Phred quality score ≤ 5) and reads with N ratio higher than 1% were filtered using the filter-fq program and then removed to produce the clean reads. Clean reads were mapped to the reference genome [22] using HISAT2 (v2.1.0). FPKM values (expected number of fragments per kilobase of transcript sequence per millions base pairs sequenced) of each unigenes were calculated using the HTSwq package (v0.6.0), which were further compared between groups using the DESeq2 R package (v3.8) to represent relative expression levels. Differences with absolute fold change of FPKM value > 2 and q value ≤0.001 were considered statistically significant [23] and these unigenes were considered differentially expressed genes (DEGs).

Ten DEGs were randomly selected from the top 200 highly expressed DEGs and their expression levels were verified by RT-qPCR. All gene-specific primers were designed using the NCBI primer designing tools (Primer3 and Primer-BLAST) to ensure their specificity to the target genes in rice. Glyceraldehyde-3-phosphate dehydrogenase (GAPCP1), which was stably expressed in all samples, was used as the internal control. The primer sequences are listed in Supplementary Table S1. cDNA was synthesized from total RNA (the same RNA samples for Illumina sequencing) using BioRT cDNA first strand synthesis kit (Bioer, Hangzhou, China) and oligo (dT) primer. RT-qPCR was carried out using BioEasy master mix (Bioer, Hangzhou, China) on a Line Gene9600 Plus qPCR machine (Bioer, Hangzhou, China). Each reaction was repeated three times as technical replicates. Three independent biological replicates were included for each treatment. Relative expression levels to GAPCP1 were analyzed using the 2^-ΔΔCT^ method. Student’s t-tests were applied to compare differences between treatments. *P* < 0.05 was considered statistically significant.

### Functional annotation and classification of DEGs

Gene ontology (GO) annotations were performed using Blast2GO v2.5 against the non-redundant (Nr) nucleotide and protein databases on National Center for Biotechnology Information (NCBI). DEGs were mapped to the KEGG (Kyoto Encyclopedia of Genes and Genomes) database for enrichment of pathways using clusterProfiler3 (v3.8). The significance of KEGG enrichment was corrected to control the false discovery rate (FDR) using the Benjamini-Hochberg (BH) method. DIAMOND software was used to blast DEGs against the Plant Resistance Gene Database (PRGdb, http://prgdb.crg.eu/) for PRG annotation with a threshold cutoff of 40% identity and 50% coverage [24, 25].

### Coexpression network analysis

Coexpression network analysis was conducted using a online tool RiceNet version 2 (https://www.inetbio.org/ricenet/, [26]). The obtained networks were visualized in cytoscape (http://www.cytoscape.org). Nodes represent genes and links (edges) indicate interaction between genes [27].

## Results and discussion

### *R. solani* infection of rice

Up to date, no rice germplasm with complete resistance to *R. solani* has been found. However, some varieties displayed slight or moderate resistance to *R. solani*, such as ZYQ8 [28], Minghui63 [29], LSBR-33 and RSB03 [9]. The so-called resistance was not stable, dependent on environmental conditions [9]. In the present study, after inoculation for 3 days, both JG and YH showed typical SB symptoms, but the size of SB spots was smaller in YH than JG (Fig. S1), indicating the timing of SB infection was slower in YH than in JG. These results were similar to previous observation on other SB-slightly resistant rice cultivar [16] and supported the moderate resistance of YH to SB. However, after 1 week, both cultivars showed severe disease symptoms and no differences were visually observed between JG and YH. These results were consistent with a previous report that the main difference between resistant and susceptible rice cultivars was the timing of responses after infection [17].

### Summary of RNA sequencing

The raw RNA-seq data of the 12 rice samples have been deposited in the NCBI with the accession number of PRJNA551731. After filtration, the total clean reads of each sample ranged from 60.95 M to 63.05 M. The Q20 values and Q30 values of each sample were higher than 96.91 and 88.50%, respectively (Supplementary Table S2).

Overall, 86% of the total clean reads could map to the genome of *O. sativa* Japonica Group (Japanese rice). Identification of novel genes/transcript isoforms is one of the major advantages of RNA-seq technology [30]. In the present study, a total of 12,244 novel transcripts were detected, including 10,162 coding transcripts and 2082 non-coding transcripts. Besides, 8964 novel isoforms and 1198 novel genes were identified. These identified novel transcripts or isoforms required further investigations in future to explore their biological functions in rice.

### DEGs and RT-qPCR validation

**Fig. 1:**
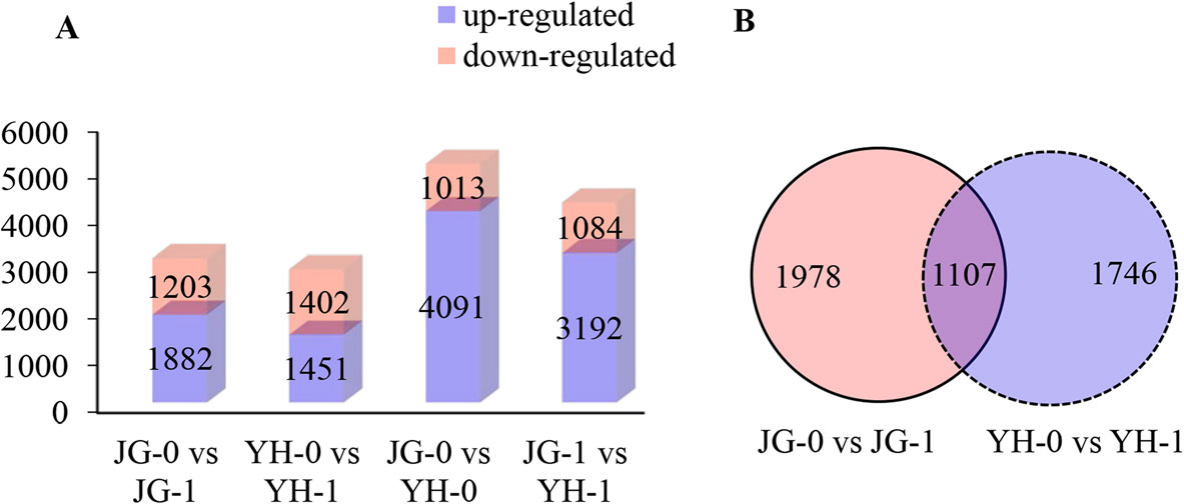
Numbers of DEGs in rice cultivars JG and YH before and after *R. solani* infection. **a** The numbers of upregulated and downregulated DEGs detected in JG and YH after *R. solani* inoculation for 3 days. **b** Venn diagram of DEGs in JG-0 vs JG-1 and YH-0 vs YH-1. JG-0 and YH-0: uninfected cultivars. JG-1 and YH-1: samples infected with *R. solani*

Before inoculation of *R. solani*, 4091 and 1013 unigenes showed significantly higher and lower expression levels in YH-0 than in JG-0, suggesting great genetic differences between these two cultivars. After infecting *R. solani*, 3192 unigenes displayed significantly higher expression levels in YH-1 than JG-1 (Fig. 1a), which might be important for the higher resistance in YH.

Compared with the corresponding uninfected samples, 1882 and 1451 unigenes were upregulated, and 1203 and 1402 unigenes were downregulated in infected JG (JG-1) and YH (YH-1), respectively (Fig. S2 and S3). Among them, 1107 DEGs were shared between comparison of JG-1 vs JG-0, and comparison of YH-1 vs YH-0 (Fig. 1b). Moreover, 241 and 223 novel genes were differentially expressed between infected and uninfected samples in JG and YH, respectively. Correlation analysis between biological replicates is shown is Fig. S4. The sample JG-0-2 showed the lowest correlation with other samples, probably because this sample showed the most severe infection symptom.

**Fig. 2:**
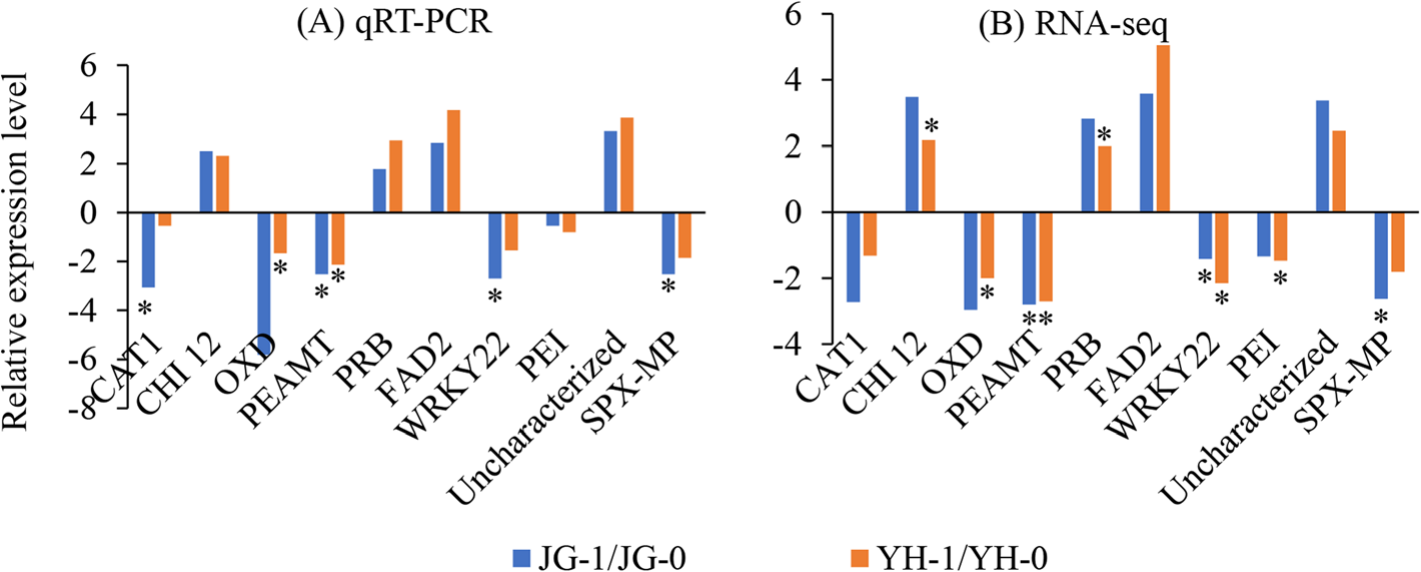
Validation of RNA-seq data via qRT-PCR. JG-0 and YH-0: uninfected cultivars. The relative expression levels represent the fold changes to the control sample. Positive numbers represent upregulation and negative number reporesent downregulation. JG-1 and YH-1: cultivars infected with *R. solani.* Gene names are listed in Table S1. *indicates significantly difference between infected and uninfected samples (*P* < 0.05)

To validate RNA-seq results, RT-qPCR was conducted on 10 unigenes. These genes were involved in plant-pathogen interaction, plant hormone signal transduction, and phenylpropanoid biosynthesis pathways. Both upregulated and downregulated genes in infected samples compared with uninfected samples were included. Melting curves of qPCR products showed unique peak for all genes, suggesting the specificity of primers. The relative expression levels of all the selected genes obtained by RT-qPCR analysis were in agreement with those calculated by FPKM values (Fig. 2), suggesting that the RNA-seq results were reliable.

### Annotation of transcription factors (TFs) and functions of WRKY TFs

**Fig. 3:**
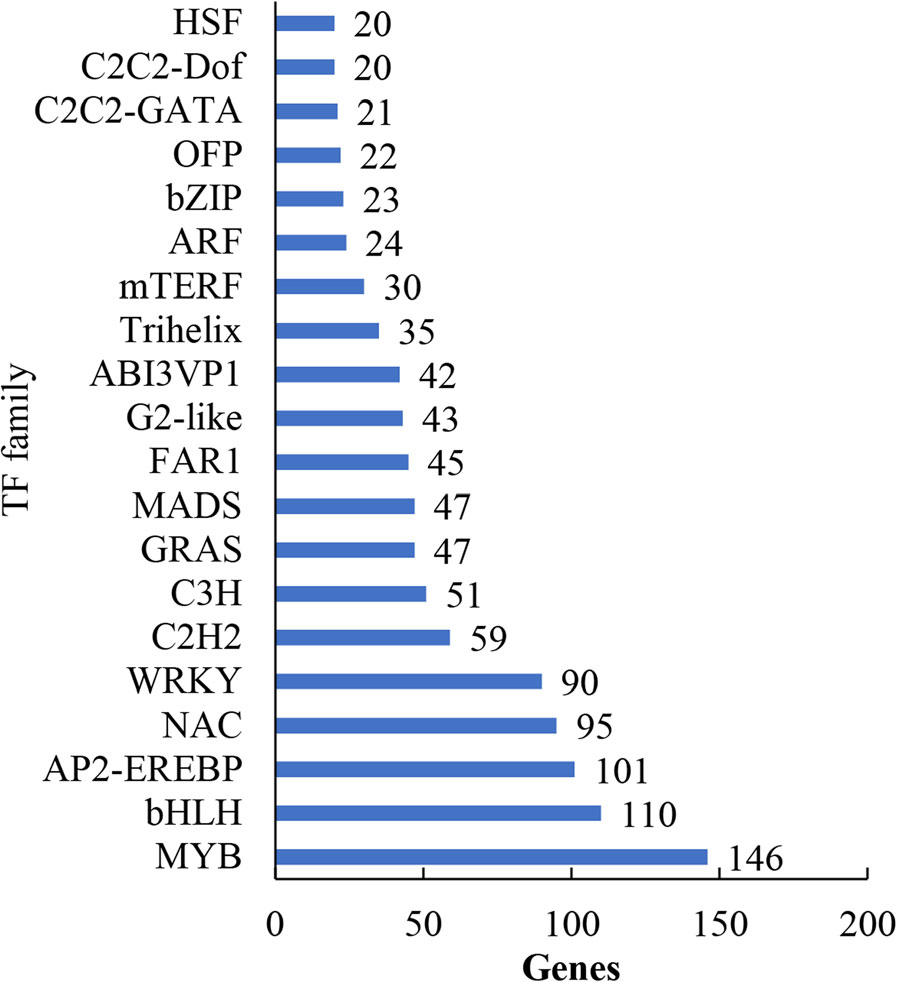
Annotation and classification of rice transcriptome against transcription factor (TF) database

Over the past two decades, molecular and genetic studies have discovered numerous TFs that are critical in regulating proper transcriptional responses when plants are infected by phytopathogens. In the present study, a total of 1364 TFs were detected in rice transcriptome, which were classified into 57 families. The top 20 of TF families are exhibited in Fig. 3. Among them, MYB (146), bHLH (110), AP2-EREBP (101), NAC (95) and WRKY (90) TF families occupied more than 39.74% of the total number of TFs (Fig. 3).

Among these TFs, WRKY is one of the most important TF families in higher plants and have been reported to widely participate in pathogen defense responses in plants. For example, WRKY44 mediated defense responses to *R. solanacearum* and *R. solani* infections in cotton [31]. Mutation of WRKY33 increased susceptibility to *Botrytis cinerea* and *Alternaria brassicicola* in *Arabidopsis* [32]. WRKY71 functioned as a transcriptional regulator upstream of NPR1 and PR1b in rice defense signaling pathways against *Xanthomonas oryzae* [33]. In the present study, WRKY22 (*P* < 0.05) was significantly downregulated in JG-1 and YH-1, compared with the control (*P* < 0.05); while WRKY33 was downregulated in YH-1, compared with YH-0 (*P* < 0.05; Table S3). Knockout of WRKY22 enhanced susceptibility to *Magnaporthe oryzae* and altered cellular responses to nonhost *Magnaporthe grisea* and *Blumeria graminis* fungi, and overexpression of WRKY22 enhanced resistant phenotypes in rice [34]. WRKY33 is a transcription factor required for resistance to necrotrophic pathogens [32]. Thus, downregulation of WRKY22 in JG and YH cultivars, and of WRKY33 in YH cultivar might be responses post infection. More interestingly, expression level of WRKY33 was 20 times higher in YH-0 than JG-0, and 3.7 times higher in YH-1 than JG-1 (Table S3). Higher expression level of WRKY33 would benefit resistance of rice to *R. solani* infection. Similarly, Zhang et al. [17] reported that WRKY24, WRKY53 and WRKY70 were more highly expressed in *R. solani*-resistant rice cultivar (TeQing) than susceptible cultivar (Lemont), which might contribute to the higher resistance to *R. solani* in TeQing cultivar. The mRNA sequences of WRKY33 in YH and JG were aligned. These two sequences were exactly the same (Supplementary Alignment File 1). The regulatory mechanisms of WRKY33 transcription in YH need further investigations.

### Annotation of plant resistance genes (PRGs)

**Fig. 4:**
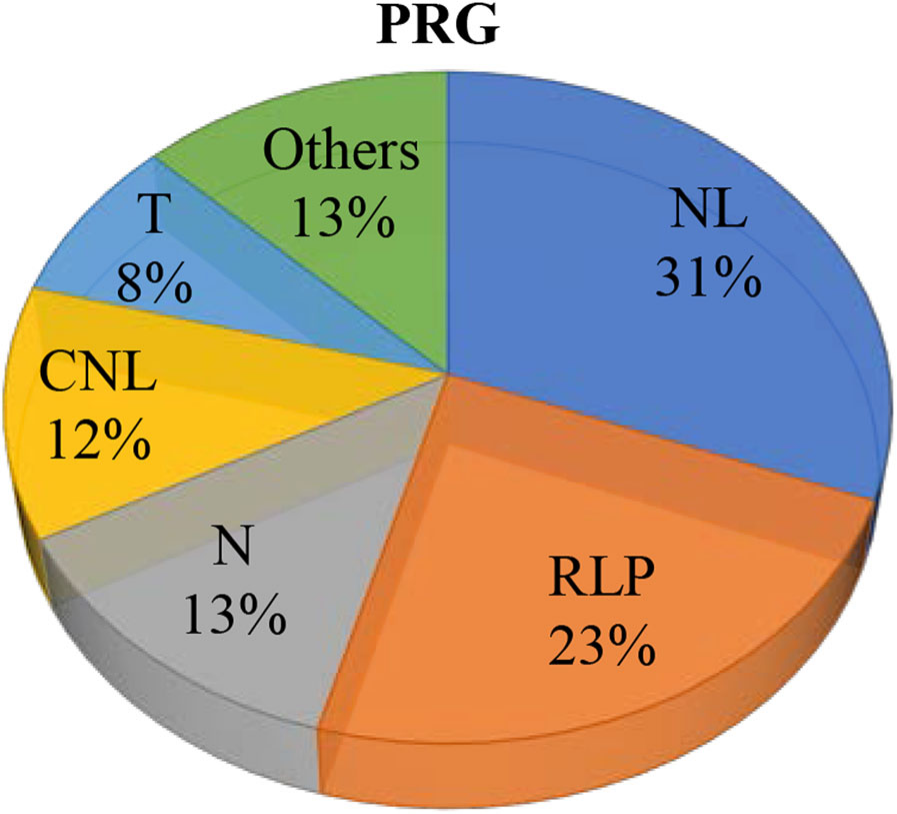
Annotation and classification of rice transcriptome against plant resistance genes (PRGs)

Plant resistance genes (PRG) can be functionally grouped into five distinct classes based on the presence of specific domains, including CNL class (containing a N-terminal coiled coil domain, a nucleotide-binding site and a leucine-rich repeat, namely CC-NBS-LRR), TNL class (containing a Toll interleukin1 receptor domain, a nucleotide-binding site and a leucine-rich repeat, namely TIR-NBS-LRR), RLP class (receptor-like protein, containing a receptor serine threonine kinase-like domain and an extracellular leucine-rich repeat), RLK class (receptor-like kinase, containing a kinase domain and an extracellular leucine-rich repeat) and “Other” class (which has no typical resistance related domains) [35]. In the present study, a total of 943 PRGs were detected in transcriptomes of both cultivars (Fig. 4). Among them, NL (292, containing NBS domain at N-terminal and LRR at the C-terminal, and lack of the CC domain), RLP (220), N (121, containing NBS domain only, lack of LRR), CNL (115), and T (76, contains TIR domain only, lack of LRR or NBS) domains occupied more than 87.38% of the total number of PRGs (Fig. 4), which have been reported to participate in responses to various abiotic stresses in different plants [36].

### Coexpression network analysis

**Fig. 5:**
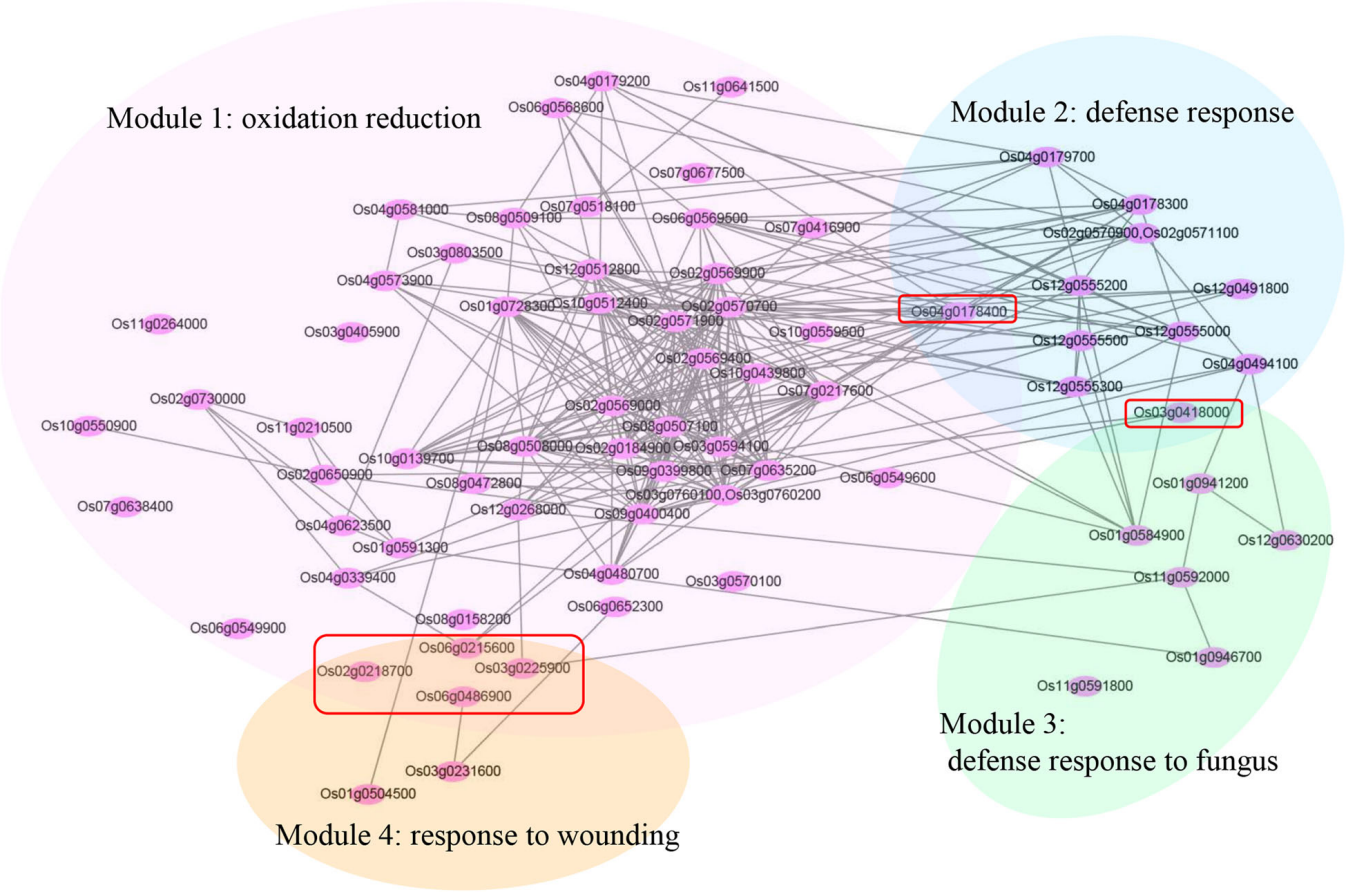
The coexpression network of genes upregulated in both infected treatments. The coexpression between two genes is indicated by an edge. Hub genes between two modules are shown in red box

Coexpression network analysis provides clues for establishing the putative functions of the genes involved in biological processes. To have better insights into the molecular responses to SB infection, coexpression network was constructed for 622 genes upregulated in both infected cultivars compared with the control. Finally, the network showed 762 edges among 225 genes. These genes were mainly associated with four modules, including “oxidation reduction”, “defense response”, “defense response to fungus” and “response to wounding”. In these modules, Os04g0178400 (cytochrome P450 mono-oxygenase gene, CYP99A3), Os03g0418000 (Chitinase 12, Cht12), Os06g0215600 (12-oxophytodienoate reductase 5, OsOPR5), Os03g0225900 (Allene oxide synthase 2, CYP74A2), Os06g0486900 (Formate dehydrogenase 2, FDH2) and Os02g0218700 (Allene oxide synthase 3, CYP74A3) were hub genes and involved in at least two modules (Fig. 5).

**Fig. 6:**
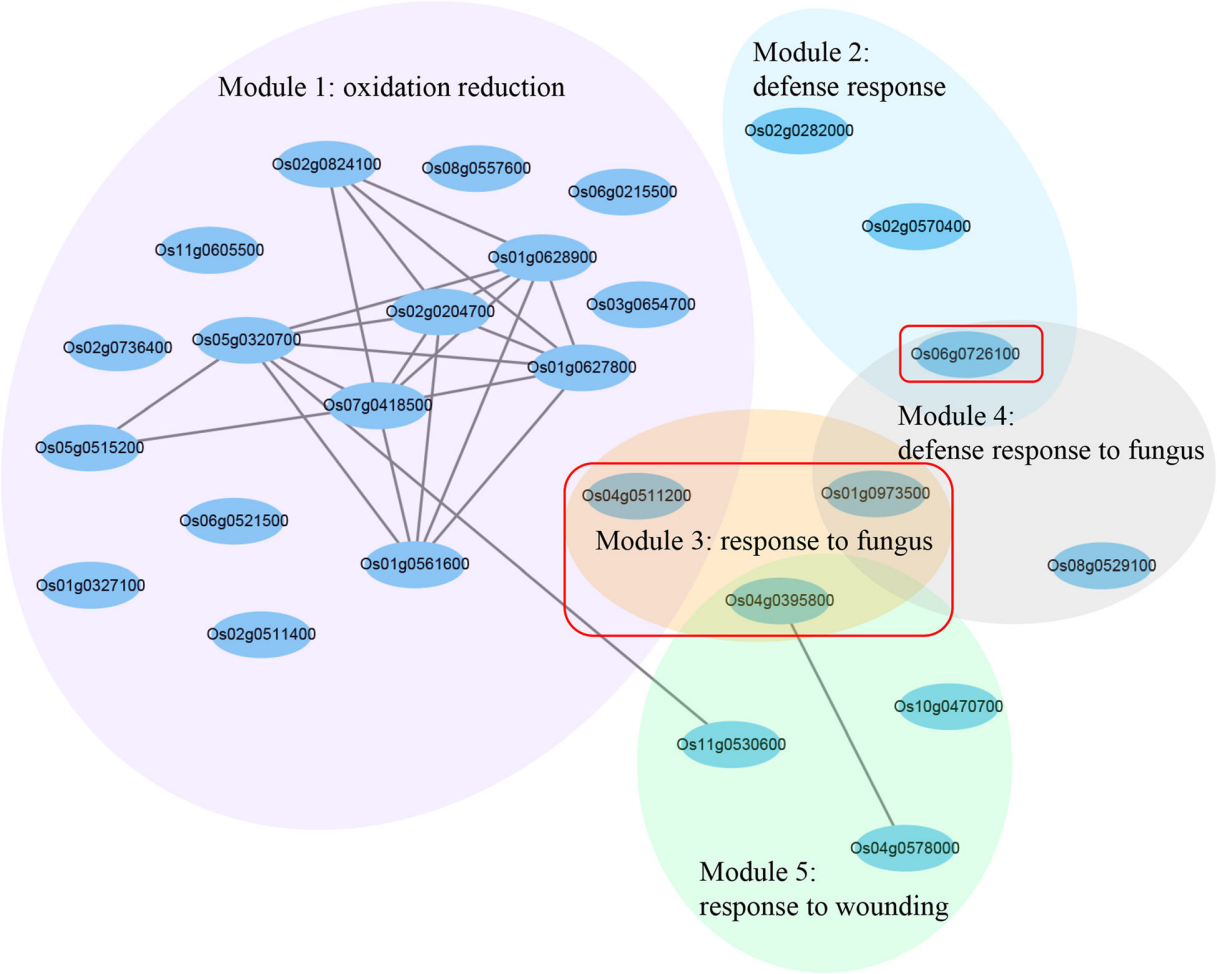
The coexpression network of gene upregulated YH-1 but not in JG-1. The coexpression between two genes is indicated by an edge. Hub genes between two modules are shown in red box

To reveal the potential mechanisms underlying resistance to SB in YH cultivar, 541 upregulated genes in YH-1 (compared with YH-0) but not in JG-1 (compared with JG-0) were subjected to coexpression analysis. The results showed that 202 genes formed 431 edges. Among them, 26 genes forming 23 edges were assigned to five modules, including “oxidation reduction”, “defense response”, “response to fungus”, “defense response to fungus” and “response to wounding” (Fig. 6). In the network, Os04g0511200 (Peroxygenase, PXG), Os04g0395800 (protein TIFY9), Os01g0973500 (Receptor-like cytoplasmic kinase 176, RLCK176) and Os06g0726100 (Chitinase 3, Cht3) were the hub genes. PXG is related to plant cytochrome P450s, which is involved in the peroxygenase pathway and contributes to antifungal properties [37]. The TIFY gene family participates in plant defense against insect feeding, wounding, pathogens and abiotic stresses [38]. OsRLCKs play important roles in plant growth, environmental stress and pathogen response [39]. The chitinase gene is the most commonly used pathogenesis-related (PR) gene and there was a significantly positive correlation between SB resistant ability and chitinase activity in transgenic plants [40]. Taken together, these genes might be candidate genes for genetic breeding of SB resistant cultivars.

### GO annotation and enrichment analyses

**Fig. 7:**
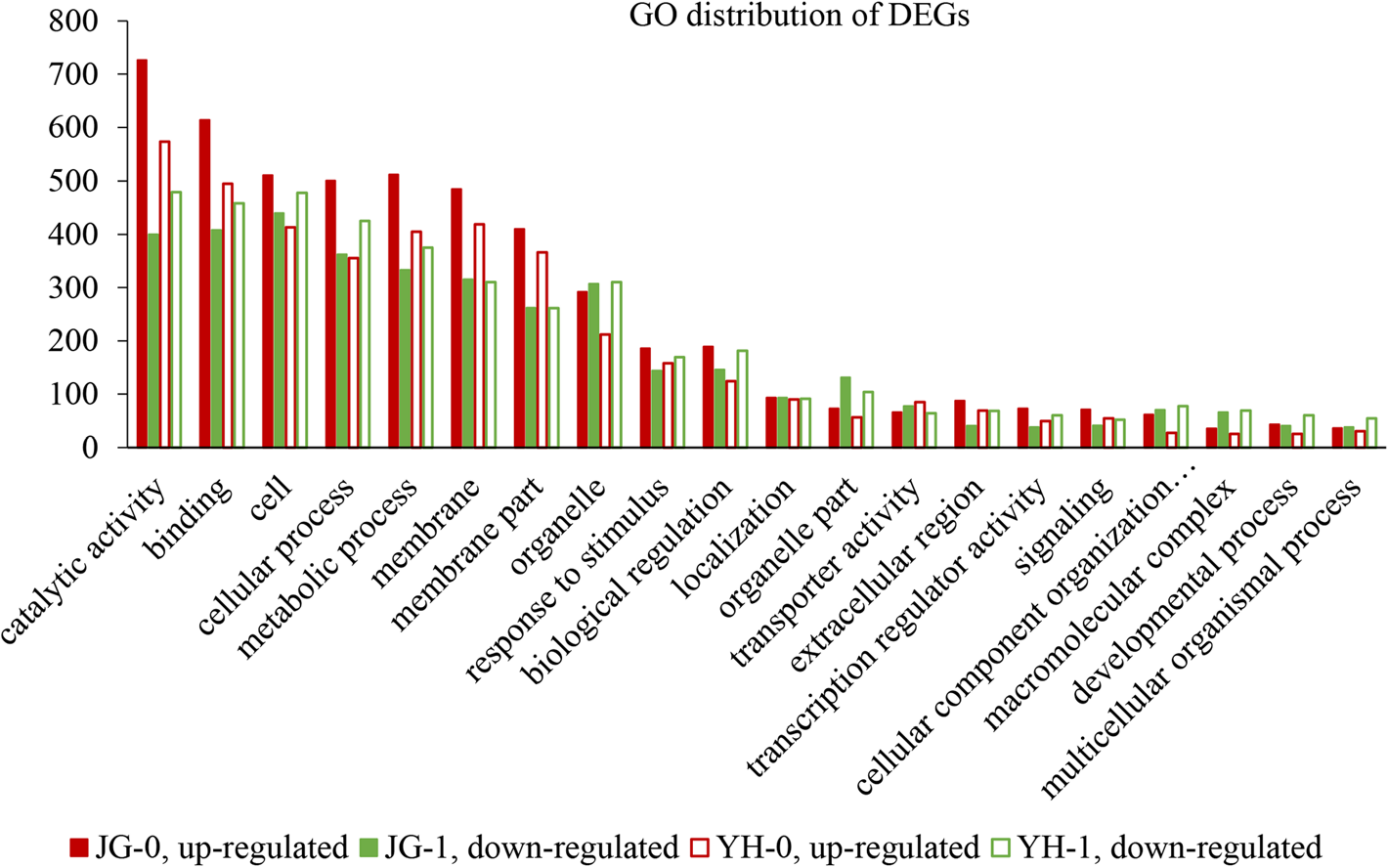
Gene ontology (GO) distribution of DEGs in comparisons between JG-0 and JG-1, between YH-0 and YH-1. JG-0 and YH-0: uninfected cultivars. JG-1 and YH-1: cultivars infected with *R. solani*

Compared with JG-0, a total of 2058 DEGs, including 1253 upregulated and 805 downregulated unigenes, in JG-1 treatment were mapped to 47 GO level 2 classes. A total of 1913 DEGs, with 988 upregulated and 925 downregulated unigenes in treatment with YH-1 in comparison to YH-0, hit 43 GO level 2 classes. Comparisons between infected and uninfected treatments showed similar distribution of GO level 2 classes in JG and YH cultivars. The top five GO level 2 classes included catalytic activity, binding, cell, cellular process and metabolic process (Fig. 7).

Enrichment of GO terms revealed that 100 and 127 GO terms were significantly enriched in comparisons between JG-0 and JG-1, YH-0 and YH-1 respectively. In both JG, the top five enriched GO terms were oxidoreductase activity (GO: 0016491), secondary metabolic process (GO: 0019748), secondary metabolite biosynthetic process (GO: 0044550), tetrapyrrole binding (GO: 0046906) and heme binding (GO:0020037); while in YH, secondary metabolite biosynthetic process (GO: 0044550) was replaced by oxidoreductase activity (GO: 0016705, acting on paired donors, with incorporation or reduction of molecular oxygen). The results suggested that these two cultivars might share general mechanisms in response to *R. solani* infection. However, there were some differences in enriched GO terms between YH and JG cultivars. The GO terms “metal ion binding” (GO: 0046872) and “cation binding” (GO: 0043169) were enriched in comparison between JG-0 and JG-1, but not in comparison between YH-0 and YH-1. Similarly, the GO term “response to stimulus” (GO: 0050896) and “response to chemical” (GO: 0048878) were enriched in YH but not in JG. “Response to stimulus” and “response to chemical” are two typical terms functioning as “defense elicitors” during fungal infection [41]. Over-representation of these two terms in YH cultivar might contribute to its resistance to SB infection. More investigations are required to clarify the underlying mechanisms.

### KEGG enrichment analysis of DEGs

**Fig. 8:**
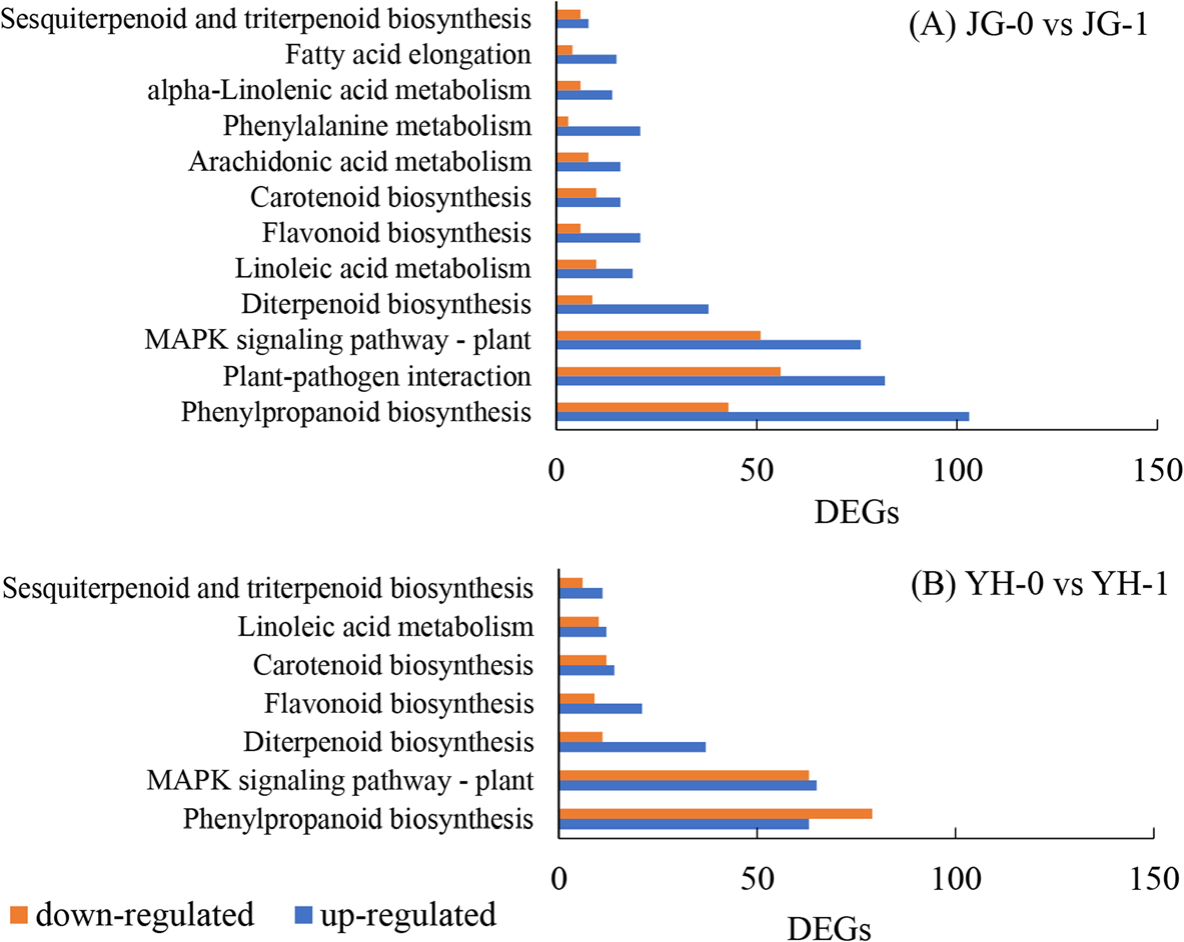
The KEGG pathway enrichment of DEGs in comparisons between JG-0 and JG-1, between YH-0 and YH-1. JG-0 and YH-0: uninfected cultivars. JG-1 and YH-1: cultivars infected with *R. solani*

KEGG annotation revealed that 1484 DEGS between JG-1 and JG-0 and 1383 DEGs between YH-1 and YH-0 were mapped to 128 and 129 KEGG pathways, respectively. Among them, 12 and 7 KEGG pathways were significantly enriched (Q value < 0.05), respectively (Fig. 8). In response to *R. solani* infection, phenylpropanoid biosynthesis, plant-pathogen interaction and MAPK signaling pathway-plant were the top three pathways enriched between JG-1 and JG-0, while, phenylpropanoid biosynthesis and MAPK signaling pathway-plant were the top two KEGG pathways between YH-1 and YH-0.

Furthermore, unigenes upregulated in YH compared with JG might also function in the higher resistance of YH than JG. These genes were also subjected to KEGG enrichment analyses. Compared with JG-0, three KEGG pathways were upregulated in YH-0, including fatty acid elongation (ko00062), sesquiterpenoid and triterpenoid biosynthesis (ko00909) and phenylpropanoid biosynthesis (ko00940). After infection, four KEGG pathways were significantly upregulated in YH-1 than JG-1, including plant-pathogen interaction (ko04626), sesquiterpenoid and triterpenoid biosynthesis (ko00909), beta-alanine metabolism (ko00410) and DNA replication (ko03030, Table 1).

**Table 1:** KEGG enrichment of genes upregulated in YH-0 than JG-0, and upregulated genes in YH-1 than JG-1

Pathway ID and name	EGN	TGN	*P* value	Q value
Upregulated genes in YH-0 than JG-0				
ko00062, Fatty acid elongation	26	1829	0.000	0.001
ko00909, Sesquiterpenoid and triterpenoid biosynthesis	19	1829	0.000	0.008
ko00940, Phenylpropanoid biosynthesis	175	1829	0.001	0.045
Upregulated genes in YH-1 than JG-1				
ko04626, Plant-pathogen interaction	144	1423	0.000	0.001
ko00909, Sesquiterpenoid and triterpenoid biosynthesis	17	1423	0.000	0.004
ko00410, beta-Alanine metabolism	15	1423	0.000	0.007
ko03030, DNA replication	29	1423	0.001	0.022

*EGN* Enriched gene number, *TGN* Total gene number

Taken these KEGG enrichment results together, the KEGG pathway phenylpropanoid biosynthesis not only significantly responded to *R. solani* infection (JG-1 vs JG-0, YH-1 vs YH-0), but also upregulated in YH-0 than JG-0. Similarly, the KEGG pathway plant-pathogen interaction was enriched in JG before and after infection, and was upregulated in YH-1 than JG-1. Thus, these two pathways might importantly contribute to the higher resistance to SB in YH than JG.

### Potential functions of phenylpropanoid biosynthesis during *R. solani* infection

**Fig. 9:**
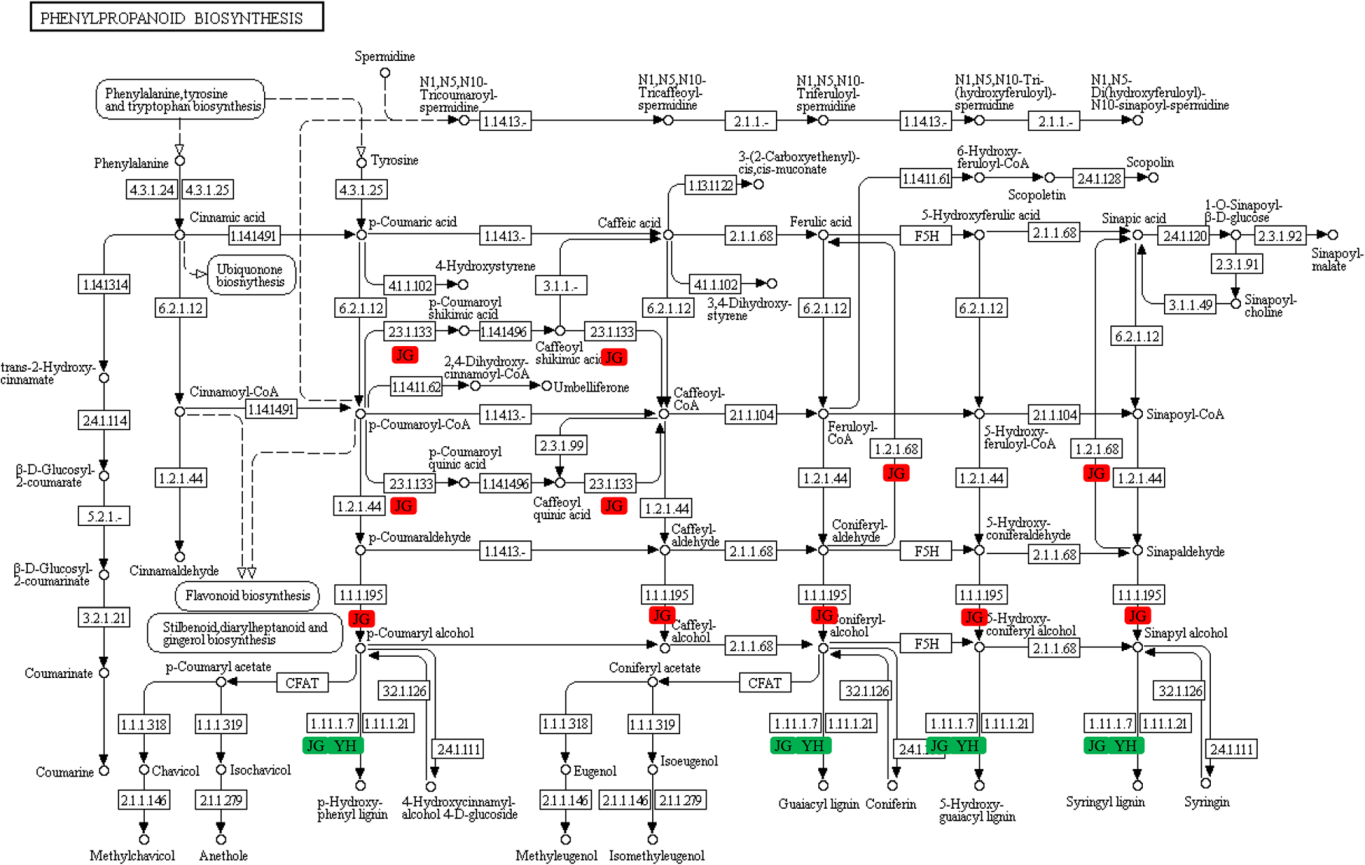
Regulations of the KEGG pathway phenylpropanoid biosynthesis. Red box: up-regulated in response to *R. solani* infection. Green box: down-regulated in response to *R. solani* infection. EC 4.3.1.24: phenylalanine ammonia-lyase, PAL; EC:4.3.1.25: phenylalanine/tyrosine ammonia-lyase, PTAL; EC 6.2.1.12: 4-coumarate--CoA ligase, 4CL; EC 3.2.1.21: beta-glucosidase; EC 1.1.1.195: cinnamyl-alcohol dehydrogenase, CAD; EC 1.2.1.44: CCR; EC 1.14.14.91: trans-cinnamate 4-monooxygenase, CYP73A; EC 1.14.11.61: Feruloyl-CoA 6-hydroxylase; EC 2.3.1.133: shikimate O-hydroxycinnamoyltransferase, HCT; EC 1.11.1.7: peroxidase, POD; EC 1.2.1.68: redox factor 1, REF1; EC 2.4.1.111: UDP–glucose: coniferyl alcohol glucosyltransferase, UGT72E

Phenylpropanoid biosynthesis converts L-Phe to diverse aromatic compounds, such as soluble phenolics, flavonoids and lignin. These compounds are key contributors to disease resistance [42]. In the present study, 146 and 142 DEGs were over-represented in phenylpropanoid biosynthesis pathway in JG and YH cultivar, respectively (Fig. 9, Table S3). These DEGs were mapped to 11 proteins in phenylpropanoid biosynthesis. Among them, cinnamyl-alcohol dehydrogenase (CAD), redox factor 1 (REF1), shikimate O-hydroxycinnamoyl transferase (HCT) were significantly upregulated in JG-1, compared with JG-0 (*P* < 0.05, Table S3). CAD and HCT are key enzymes in lignin biosynthesis pathway, which is a component of the cell wall and is essential for pathogen resistance in plants. So far, 12 CAD homologues have been identified in the genomes of rice [43]. At the transcriptional level, CAD genes in tea plants may play a role in defense against insects, pathogens and adaptation to abiotic stresses [44]. As a bifunctional enzyme [45], downregulation of HCT decreased lignin content in plants [46–48]. REF1 is a bifunctional protein that is transcriptionally up-regulated in response to oxidative stresses [49]. In vivo, REF1 expression inversely correlates with susceptibility to reperfusion injury [50]. Upregulation of these genes in JG-1 than in JG-0 revealed the activation of phenylpropanoid biosynthesis, which might promote resistance to *R. solani* infection. No significant differences in expression levels of these genes in YH were detected before and after infection, but their expression levels in YH-1 were similar to those in JG-1 (Table S3). These results were consistent with the later responses of resistant cultivar than susceptible cultivar during infection [17].

Peroxidase (POD) is known to be induced by various pathogens infection. Infection of rice leaves by *Xanthomonas oryzae* strongly induced a POD isoform and reduced access of the pathogen to membrane [51]. In the present study, POD was downregulated in infected JG-1 and YH-1, compared with the uninfected controls (*P* < 0.05, Table S3), probably a phenomenon of post infection.

Trans-cinnamate 4-monooxygenase (CYP73A, C4H) participates in the biosynthesis of phytoalexins [52], which help plants to resist fungal infections [53]. In the present study, C4H revealed higher expression levels in infected YH and JG cultivars than in the controls, suggesting that this gene might be important to resistance to *R. solani*. More importantly, C4H showed 111 times higher expression level in YH-0 than JG-0, 101 times higher in YH-1 than JG-1. Thus, it might be a potential candidate to explain the higher resistance of YH than JG. The mRNA sequences of C4H in JG and YH were aligned. Two nucleotide mutations were observed at the 3′ untranslational region (Supplementary Alignment File 2). Whether these two mutations regulate the transcription of C4H should be further investigated.

### Potential functions of plant-pathogen interaction pathway during *R. solani* infection

The KEGG pathway plant-pathogen interaction was significantly enriched in DEGs between JG-0 and JG-1. It has been reported that the inducible plant defense response to pathogens is multilayered and at least two stages are involved [54]. At the first stage, plant pattern recognition receptors (PRRs) trigger the recognition of pathogen-associated molecular patterns (PAMPs), resulting in PAMP-triggered immunity (PTI). The second stage is initiated by the recognition of pathogen virulence proteins (effectors) or their activities by plant disease resistance genes and the consequence is effector-triggered immunity (ETI) [55].

**Fig. 10:**
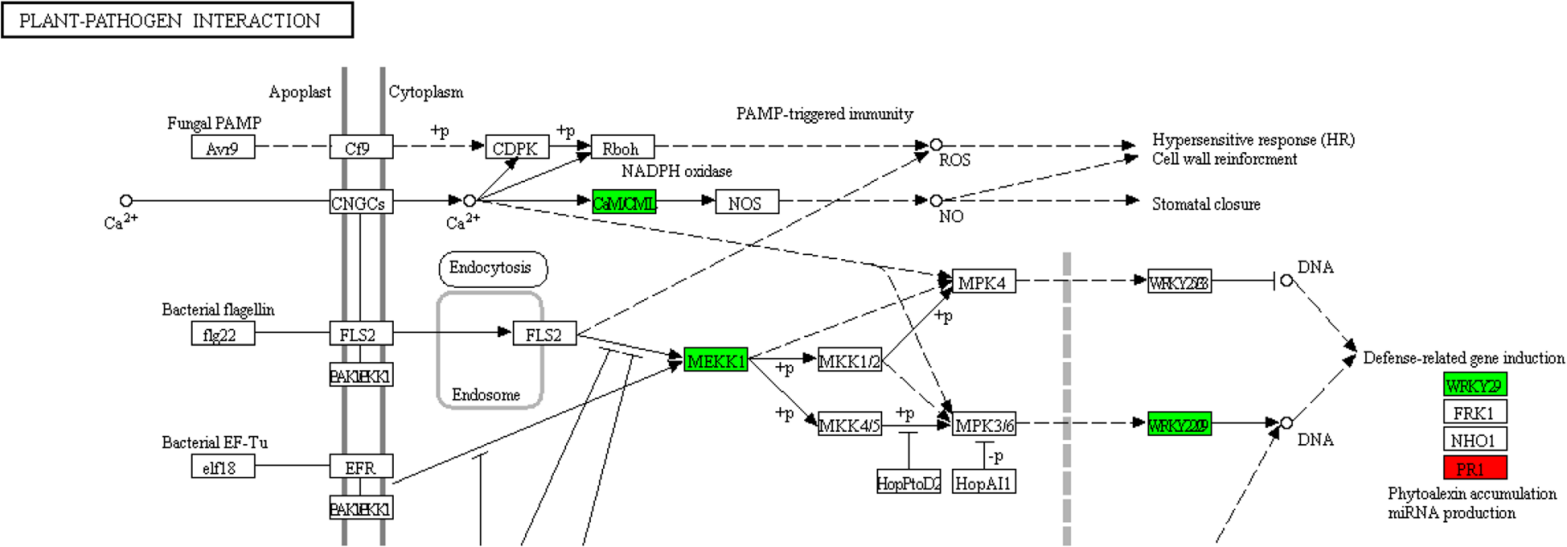
Regulations of plant-pathogen interaction in comparisons between JG-0 and JG-1. Red box: upregulated in JG-1 compared with JG-0. Green box: downregulated in JG-1compared with JG-0

In the present study, Calmodulin (CaM) and MEKK1 (*P* < 0.05, Table S3) were significantly downregulated for 1.73 and 2.87 times in JG-1, compared with JG-0 (Fig. 10 and Table S3). CaM plays a crucial role in plant defense signaling [56]. MEKK1 also functions in immune responses in plants [57]. Downregulation of these genes in JG-1 indicated that JG failed to initiate the plant-pathogen interaction for resistance.

Pathogenesis-related protein 1 (PR1) is tightly correlated with the onset of defense responses against a variety of fungal, viral and bacterial pathogens [58]. The decreased expression of PR1 and POD showed increased susceptibility to *Magnaporthe oryzae* [59] and increased PR1 level enhanced resistance to the virulent pathogen *Pseudomonas syringae* in tomato [60]. Transgenic plants overexpressing the PR-1a gene exhibited increased tolerance to the oomycete pathogens [61], whereas no resistance of PR-1 was evidenced against virus infection in tobacco plants [62]. In present study, PR1 was significantly upregulated in JG-1, compared with JG-0 (*P* < 0.05; Table S3), similar to Sarowar et al. [63]. These results indicated that JG cultivar also increased PR1 expression level after SB infection. Moreover, PR1 regulates the accumulation of phytoalexins [64]. Upregulation of PR1 was consistent with change of C4H expression in the present study, both of which regulate biosynthesis of phytoalexins.

**Fig. 11:**
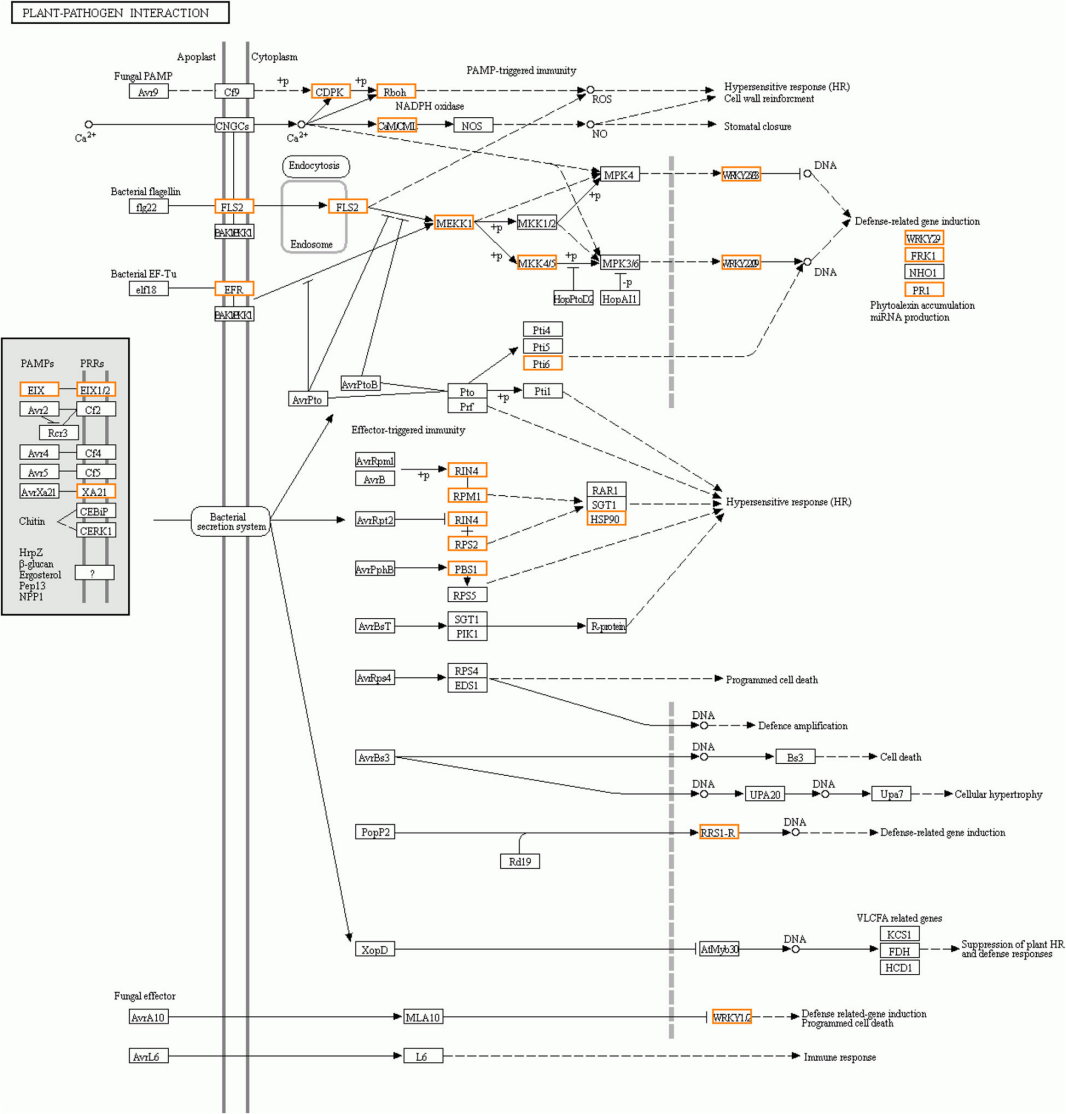
Regulations of plant-pathogen interaction in comparisons between YH-1 and JG-1. Orange box: upregulated in YH-1 compared with JG-1

KEGG analysis of upregulated genes in YH-1 than JG-1 significantly enriched the plant-pathogen interaction pathway (Table 1). These upregulated genes encode 23 proteins, which occupied approximately half of proteins in this pathway (Fig. 11). Undoubtedly, the entire plant-pathogen interaction pathway showed a higher activation status in YH than JG after *R. solani* infection. Considering the importance of plant-pathogen interaction pathway in defense against fungal pathogens [65], the higher activation status of this pathway in YH could explain its higher resistance to *R. solani* than JG.

### Potential functions of MAPK signaling pathway-plant during *R. solani* infection

**Fig. 12:**
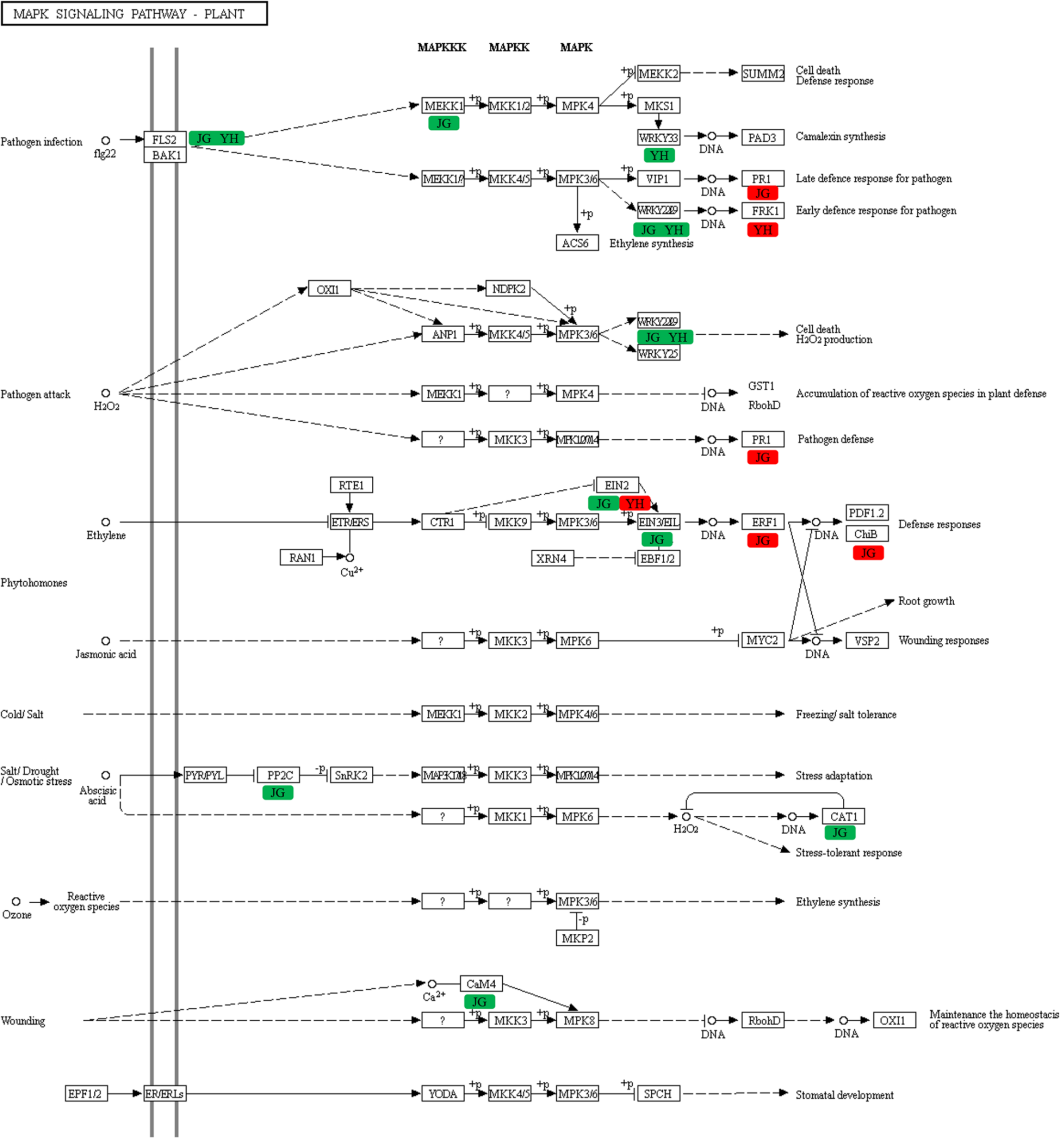
Regulations of MAPK signaling pathway-plant in comparisons between JG-0 and JG-1, between YH-0 and YH-1. Red box: up-regulated in response to *R. solani* infection. Green box: down-regulated in response to *R. solani* infection

The mitogen-activated protein kinases (MAPK) signaling pathway participates in fundamental cellular processes in response to external stimuli in all eukaryotes. Plant MAPK cascades play pivotal roles in the signaling of plant defense against pathogen attack [66]. When cells were stimulated by external signals, MAPK pathway was activated and then transduced and amplified the signals by phosphorylating MAPK kinase kinase (MAPKKK), MAPK kinase (MAPKK) and MAPK sequentially. Then, the MAPK proteins regulated downstream functional genes to initiate metabolic responses [67]. Approximately 75 putative MAPKKKs have been reported in rice [68], which exhibit differential regulation under stresses [69]. In the present study, 127 and 128 DEGs were enriched in MAPK signaling pathway in comparison between JG-1 and JG-0, between YH-1 and YH-0, respectively. Among them, four genes were upregulated and eight genes were downregulated in infected JG-1, compared with JG-0 (*P* < 0.05, Table S3). Two genes were significantly upregulated and three genes were significantly downregulated in infected YH-1, compared with YH-0 (*P* < 0.05, Fig. 12, Table S3). Greater number of *R. solani*-induced downregulated genes in JG than YH cultivar in the MAPK signaling pathway probably enhanced the susceptibility of JG cultivar to *R. solani* infection.

In the MAPK signaling pathway, WRKY transcription factor 22 (WRKY22) and senescence-induced receptor (FRK1) mediate early response to pathogens, while transcription factors VIP1 and PR1 regulate late defense response to pathogens. In the present study, FRK1 was significantly upregulated in YH cultivar (*P* < 0.05) and PR1 was significantly upregulated in JG cultivar (*P* < 0.05 for JG) after infection, consistent with Andreasson et al. [60] and the phenotypical observation in the present study.

Ethylene is a plant hormone regulated by the MAPK pathway and participates in disease resistance responses in rice [70]. Ethylene-insensitive protein 2 and 3 (EIN2 and EIN3) act downstream of the MAPK pathway [71] and activate ethylene response factor (ERF) to initiate ethylene-mediated responses, such as induction of endochitinase B (ChiB) [72]. Chitinases degrade chitin, a beta-1,4-linked polymer of N-acetyl-D-glucosamine that often comprises the cell walls of fungal pathogens and the exoskeletons of arthropods [73]. Induction of chitinase represented a later response to pathogen [74]. In the present study, ERF1 and ChiB were significantly upregulated in JG cultivar, representing a later response to *R. solani*. These results were consistent with the predicted responses of upregulated PR1 in JG-1. The sequences of two EIN2 isoforms did not differ between JG and YH (Supplementary Alignment File 3 and 4). However, EIN2 was significantly upregulated for 1.3 times in YH-1 than YH-0 (*P* < 0.05), expression level of EIN2 was 2.3 times higher in YH-0 than JG-0, and 7.7 times higher in YH-1 than JG-1 (Table S3). These results suggested the higher background level of EIN2 in YH cultivar than in JG cultivar. Thus, EIN2 might also be a candidate gene involved in resistance of YH to *R. solani* infection.

## Conclusions

The present study compared the transcriptome changes of YH (resistant) and JG (susceptible) cultivars before and after *R. solani* infection. The results showed that YH and JG shared the general molecular responses to *R. solani* infection. Expression levels of C4H, EIN2, WRKY33 and the KEGG pathway plant-pathogen interaction were not only changed in response to *R. solani* infection in rice cultivars, but were also significantly upregulated in YH-1 than JG-1, suggesting these genes might contribute to higher resistance of YH to *R. solani* than JG and might be potential target genes for molecular breeding of *R. solani*-resistant rice cultivars.

## Supplementary information

**Additional file 1: Figure S1** Symptoms of sheath bright in YH and JG rice cultivars after 3 days.

**Additional file 2: Figure S2** The MA plot of DEGs in JG (A) and YH (B). X-axis represents A value (average expression level after log2 conversion), Y-axis represents M value (difference multiple after log2 conversion). Red represents up-regulated DEGs, blue represents down-regulated DEGs, and gray represents no significant changes

**Additional file 3: Figure S3** The Volcano plot of DEGs in JG (A) and YH (B). X-axis represents the difference multiple value after conversion of log2, Y-axis represents the significance value after conversion of -log10. Red represents the up-regulated DEGs, blue represents the down-regulated DEGs, and gray represents no significant changes.

**Additional file 4: Figure S4** The correlation between biological replicates.

**Additional file 5: Supplementary Alignment file 1** Sequence alignment of WRKY33 gene between JG and YH. * indicates identical position.

**Additional file 6: Supplementary Alignment file 2** Sequence alignment of C4H gene between JG and YH. * indicates identical position. Blue line: stop codon ATG. Red line: mutations.

**Additional file 7: Supplementary Alignment file 3** Sequence alignment of EIN2 isoform 1 gene between JG and YH. * indicates identical position.

**Additional file 8: Supplementary Alignment file 4** Sequence alignment of EIN2 isoform 2 gene between JG and YH. * indicates identical position.

**Additional file 9: Table S1**. Primers for real-time quantitative PCR of selected DEGs. **Table S2**. Summary of RNA sequencing results. **Table S3**. FPKM values of selected DEGs. Data show mean ± standard error of FPKM values. * indicates significantly difference between infected and uninfected samples (*P* < 0.05).

## Data Availability

The raw RNA-seq data of the 12 rice samples have been deposited in the NCBI with the accession number of PRJNA551731.

## Abbreviations

CAD: Cinnamyl-alcohol dehydrogenase [EC:1.1.1.195]
CaM: Calmodulin
ChiB: Endochitinase B
DEGs: Differentially expressed genes
EIN2: Ethylene insensitive 2
EIN3: Ethylene insensitive 3
ERF: Ethylene response factor
ETI: Effector-triggered immunity
HCT: Shikimate O-hydroxycinnamoyltransferase [EC:2.3.1.133]
MAPK: Mitogen-activated protein kinases
MEKK1: Mitogen-activated protein kinase kinase kinase
PAMPs: Pathogen-associated molecular patterns
POD: Peroxidase [EC:1.11.1.7]
PR: Pathogenesis-related protein
PRGs: Plant Resistance Genes
PRR: Pattern-Recognition Receptor
PTI: PAMP-triggered immunity
QTLs: Quantitative trait loci
REF1: Coniferyl-aldehyde dehydrogenase [EC:1.2.1.68]
SB: Sheath blight
TFs: Transcription Factors
