# Supplementary figures and images for: Comparison of leaf transcriptome in response to Rhizoctonia solani infection between resistant and susceptible rice cultivars
Source: BMC Genomics. 2020 Mar 19;21:245. doi: 10.1186/s12864-020-6645-6 (PMC7081601; doi:10.1186/s12864-020-6645-6)

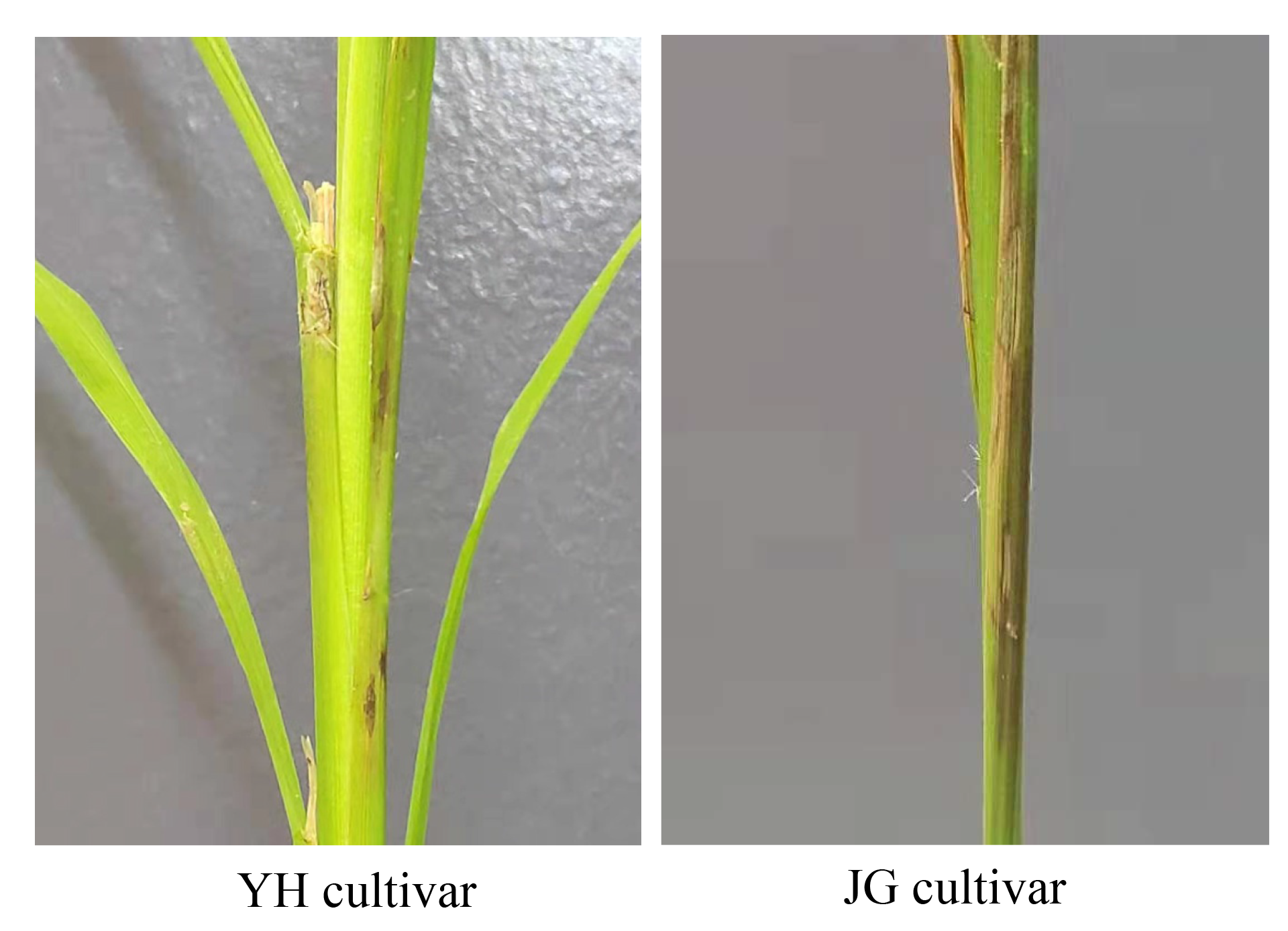

Supplement: Supplementary file 1 — Additional file 1: Figure S1 Symptoms of sheath bright in YH and JG rice cultivars after 3 days. [file 12864_2020_6645_MOESM1_ESM.tif]

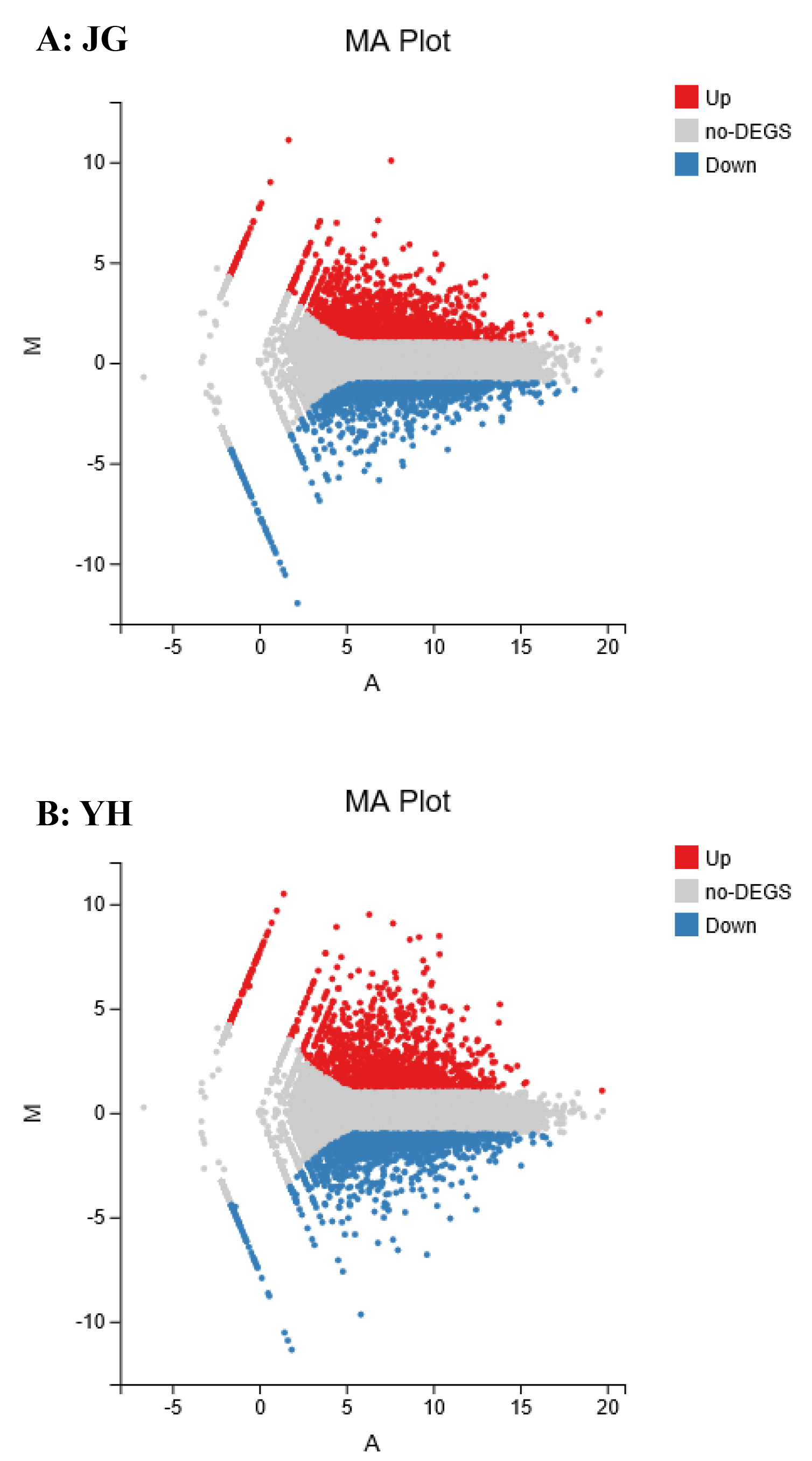

Supplement: Supplementary file 2 — Additional file 2: Figure S2 The MA plot of DEGs in JG (A) and YH (B). X-axis represents A value (average expression level after log2 conversion), Y-axis represents M value (difference multiple after log2 conversion). Red represents up-regulated DEGs, blue represents down-regulated DEGs, and gray represents no significant changes [file 12864_2020_6645_MOESM2_ESM.tif]

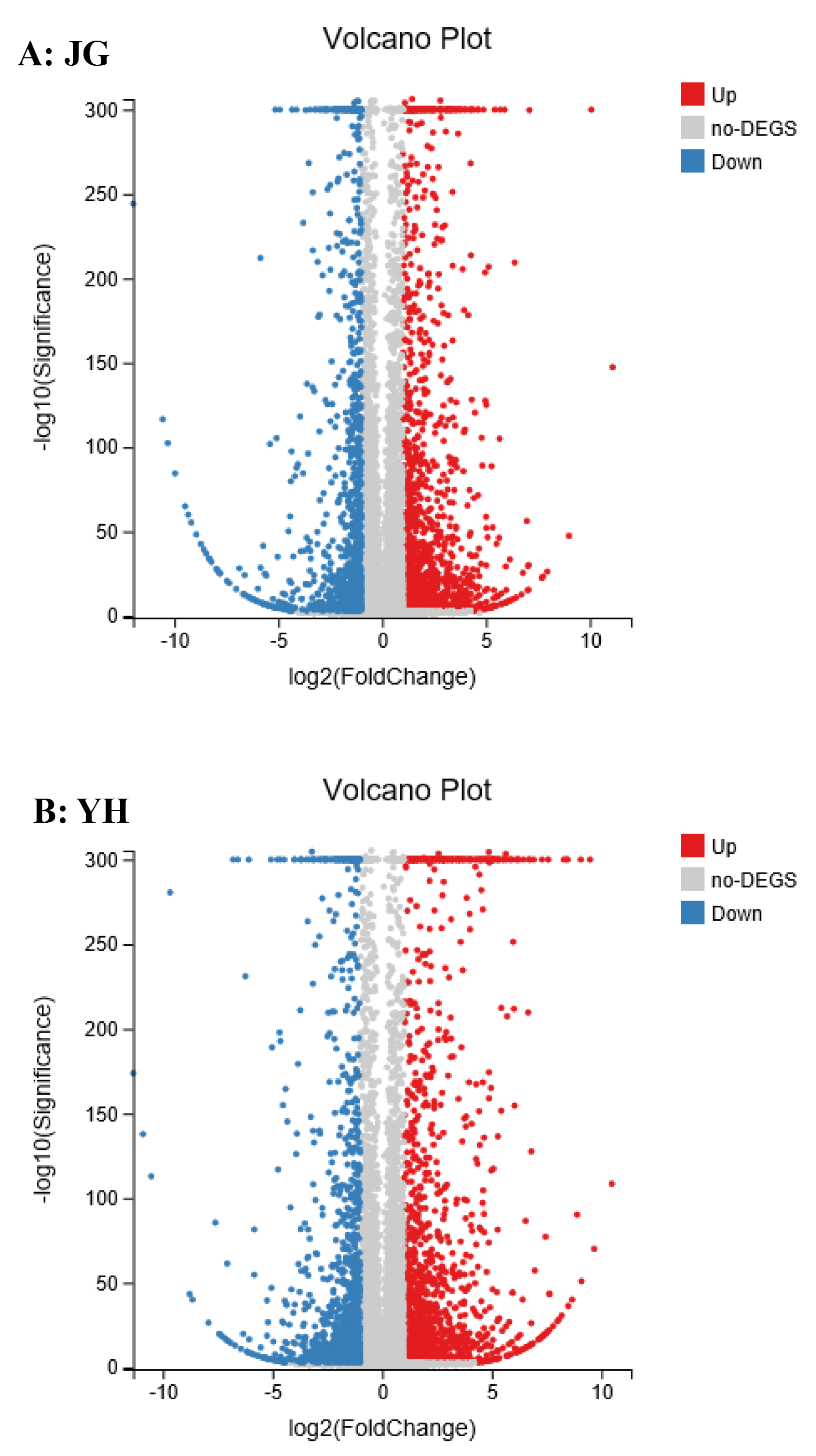

Supplement: Supplementary file 3 — Additional file 3: Figure S3 The Volcano plot of DEGs in JG (A) and YH (B). X-axis represents the difference multiple value after conversion of log2, Y-axis represents the significance value after conversion of -log10. Red represents the up-regulated DEGs, blue represents the down-regulated DEGs, and gray represents no significant changes. [file 12864_2020_6645_MOESM3_ESM.tif]

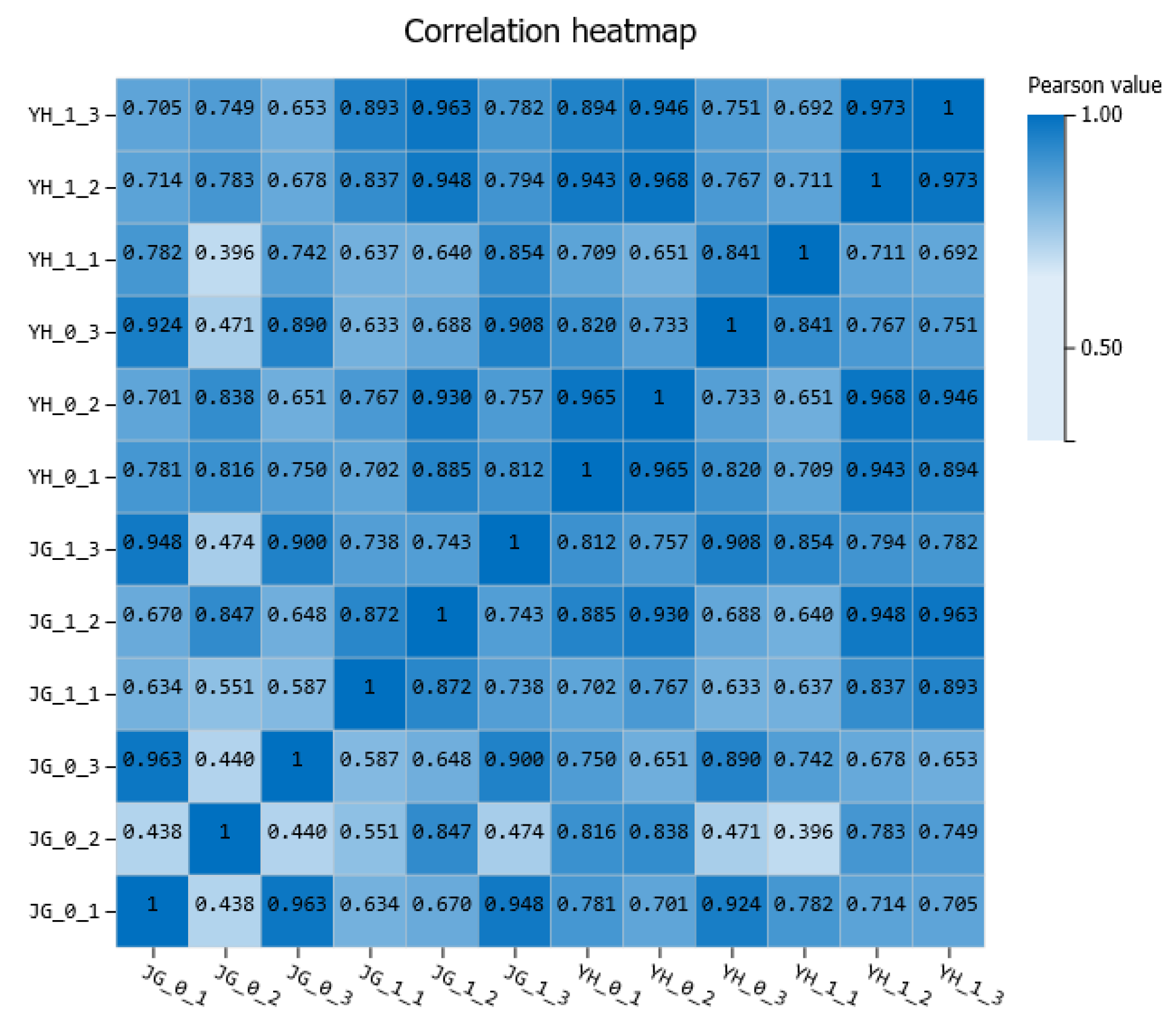

Supplement: Supplementary file 4 — Additional file 4: Figure S4 The correlation between biological replicates. [file 12864_2020_6645_MOESM4_ESM.tif]
