# Supplementary material for: Comparison of leaf transcriptome in response to Rhizoctonia solani infection between resistant and susceptible rice cultivars
Source: BMC Genomics. 2020 Mar 19;21:245. doi: 10.1186/s12864-020-6645-6 (PMC7081601; doi:10.1186/s12864-020-6645-6)
Supplement: Supplementary file 5 — Additional file 5: Supplementary Alignment file 1 Sequence alignment of WRKY33 gene between JG and YH. * indicates identical position. [file 12864_2020_6645_MOESM5_ESM.pdf]

```
YH        GCTACAGCTACAGAAATTATTCGATCCGATCGATCTCCATTAATTATCCAACGACGACAC        60
JG        GCTACAGCTACAGAAATTATTCGATCCGATCGATCTCCATTAATTATCCAACGACGACAC        60
          ************************************************************

YH        GACGACGCAGCGGCAGCGATCGATCGATGGAGGAGGCCTACTGCATGATGATGGTGGGGA        120
JG        GACGACGCAGCGGCAGCGATCGATCGATGGAGGAGGCCTACTGCATGATGATGGTGGGGA        120
          ************************************************************

YH        GGGAGAGGGAGCTCGTGGCTGAGCTGCGTCACCTCCTCTTCCCTTCTCCGTCTCCCACTC        180
JG        GGGAGAGGGAGCTCGTGGCTGAGCTGCGTCACCTCCTCTTCCCTTCTCCGTCTCCCACTC        180
          ************************************************************

YH        CGACCACCCCTGCGAGCCACTCTACTACGGCGCTCGCCGGCGACGGCGAGTGCTGCTTGC        240
JG        CGACCACCCCTGCGAGCCACTCTACTACGGCGCTCGCCGGCGACGGCGAGTGCTGCTTGC        240
          ************************************************************

YH        CGCCAGGGCTGACAACGACGACGACGGTGTCCGGTGGAGGACGGCGGCGGGGGAGGAAGA        300
JG        CGCCAGGGCTGACAACGACGACGACGGTGTCCGGTGGAGGACGGCGGCGGGGGAGGAAGA        300
          ************************************************************

YH        GAGTCAACCGCGACAATGATAATGTCAAGCTGCTTCTGCAGGCAGATGATGATCAGGAGG        360
JG        GAGTCAACCGCGACAATGATAATGTCAAGCTGCTTCTGCAGGCAGATGATGATCAGGAGG        360
          ************************************************************

YH        CCGTAATAGCTGATCATGGCGATGCAAATGCCAAGCCTCTTCCTAATTTCACCAAAACAA        420
JG        CCGTAATAGCTGATCATGGCGATGCAAATGCCAAGCCTCTTCCTAATTTCACCAAAACAA        420
          ************************************************************

YH        GAAGGAGGAAGCAGCAGGCGACGACATCAACAATGGTAACGACGGTGCCAGATTTCGATG        480
JG        GAAGGAGGAAGCAGCAGGCGACGACATCAACAATGGTAACGACGGTGCCAGATTTCGATG        480
          ************************************************************

YH        GGTATCAATGGAGGAAGTATGGTCAGAAGCAAATTGAAGGTGCCATGTACCCCAGGAGCT        540
JG        GGTATCAATGGAGGAAGTATGGTCAGAAGCAAATTGAAGGTGCCATGTACCCCAGGAGCT        540
          ************************************************************

YH        ACTACCGGTGCACCAACAGCACAAACCAGGGCTGCCTCGCCAAGAAGACGGTGCAGCGCA        600
JG        ACTACCGGTGCACCAACAGCACAAACCAGGGCTGCCTCGCCAAGAAGACGGTGCAGCGCA        600
          ************************************************************

YH        ATGGCGGCGGCGGAGCAGCAGGGTACACGGTGGCCTACATCTCAGAGCATACTTGCAAGT        660
JG        ATGGCGGCGGCGGAGCAGCAGGGTACACGGTGGCCTACATCTCAGAGCATACTTGCAAGT        660
          ************************************************************

YH        CCATCGAACCATCCCTGCCTCCAGTCATCCTCGACACCACCGTCCGTACTACCAACAACC        720
JG        CCATCGAACCATCCCTGCCTCCAGTCATCCTCGACACCACCGTCCGTACTACCAACAACC        720
          ************************************************************

YH        ACCAGCAGCCTGCAGCTGCTGAATCACCGGCGGCGACATCGTCGTCGTCGTCGAATATGG        780
JG        ACCAGCAGCCTGCAGCTGCTGAATCACCGGCGGCGACATCGTCGTCGTCGTCGAATATGG        780
          ************************************************************

YH        TCATGACGAGTAGCGAGACTGGTAATTGGAGCGGTCAGCACGGCGCCTACGCATGCCGAC        840
JG        TCATGACGAGTAGCGAGACTGGTAATTGGAGCGGTCAGCACGGCGCCTACGCATGCCGAC        840
          ************************************************************

YH        AGATGATCGCCGCCGATGAAGAGTACTGCTGCTGGGACACTCCGGCGACGACGACGACCA        900
JG        AGATGATCGCCGCCGATGAAGAGTACTGCTGCTGGGACACTCCGGCGACGACGACGACCA        900
          ************************************************************

YH        CCTCTGGCTCTAATGGTGGTAACAGTACTTGTGCTGAGGATATAGAATTATTGAGCAGGC        960
JG        CCTCTGGCTCTAATGGTGGTAACAGTACTTGTGCTGAGGATATAGAATTATTGAGCAGGC        960
          ************************************************************

YH        CGATCAGGTCGCCGATGCACATCGCGGCGGAAGGGAATTGGATGGACGATTTGCTCCTTG        1020
JG        CGATCAGGTCGCCGATGCACATCGCGGCGGAAGGGAATTGGATGGACGATTTGCTCCTTG        1020
```

```
          ************************************************************

YH        TTACTGACGGCCTTATCGTTATATCACCCATTTCCTCACTTAATTTCTAGATGCACATCT      1080
JG        TTACTGACGGCCTTATCGTTATATCACCCATTTCCTCACTTAATTTCTAGATGCACATCT      1080
          ************************************************************

YH        CTCTGCTATCTCTGCTATATGTATAAGATGTTGCTTATGAATTTTGCATGATGGGCCAAC      1140
JG        CTCTGCTATCTCTGCTATATGTATAAGATGTTGCTTATGAATTTTGCATGATGGGCCAAC      1140
          ************************************************************

YH        TC        1142
JG        TC        1142
          **
```
