# Supplementary material for: Comparison of leaf transcriptome in response to Rhizoctonia solani infection between resistant and susceptible rice cultivars
Source: BMC Genomics. 2020 Mar 19;21:245. doi: 10.1186/s12864-020-6645-6 (PMC7081601; doi:10.1186/s12864-020-6645-6)
Supplement: Supplementary file 6 — Additional file 6: Supplementary Alignment file 2 Sequence alignment of C4H gene between JG and YH. * indicates identical position. Blue line: stop codon ATG. Red line: mutations. [file 12864_2020_6645_MOESM6_ESM.pdf]

```
YH_G4H      CCCAAACTCGTCTTCTCCACCGGAGCAGAGCAGCGGCGGCGGCAATGGACGCCCTCCTCG      60
JG_G4H      CCCAAACTCGTCTTCTCCACCGGAGCAGAGCAGCGGCGGCGGCAATGGACGCCCTCCTCG      60
            ************************************************************

YH_G4H      TGGAGAAGGTCCTCCTGGGCCTGTTCGTGGCGGCGGTGCTGGCCCTAGTGGTGGCCAAGC      120
JG_G4H      TGGAGAAGGTCCTCCTGGGCCTGTTCGTGGCGGCGGTGCTGGCCCTAGTGGTGGCCAAGC      120
            ************************************************************

YH_G4H      TCACCGGGAAGAGGCTCCGCCTCCCGCCCGGCCCCGCCGGCGCGCCCATCGTCGGCAACT      180
JG_G4H      TCACCGGGAAGAGGCTCCGCCTCCCGCCCGGCCCCGCCGGCGCGCCCATCGTCGGCAACT      180
            ************************************************************

YH_G4H      GGCTCCAGGTCGGCGACGACCTCAACCACCGCAACCTGATGGCGCTGGCGCGGCGGTTCG      240
JG_G4H      GGCTCCAGGTCGGCGACGACCTCAACCACCGCAACCTGATGGCGCTGGCGCGGCGGTTCG      240
            ************************************************************

YH_G4H      GCGACATCCTCCTCCTCCGCATGGGCGTCCGCAACCTGGTGGTGGTGTCCAGCCCGGACC      300
JG_G4H      GCGACATCCTCCTCCTCCGCATGGGCGTCCGCAACCTGGTGGTGGTGTCCAGCCCGGACC      300
            ************************************************************

YH_G4H      TCGCCAAGGAGGTGCTCCACACCCAGGGCGTCGAGTTCGGCTCCCGCACCCGCAACGTGG      360
JG_G4H      TCGCCAAGGAGGTGCTCCACACCCAGGGCGTCGAGTTCGGCTCCCGCACCCGCAACGTGG      360
            ************************************************************

YH_G4H      TGTTCGACATCTTCACCGGGAAGGGGCAGGACATGGTGTTCACCGTGTACGGCGACCACT      420
JG_G4H      TGTTCGACATCTTCACCGGGAAGGGGCAGGACATGGTGTTCACCGTGTACGGCGACCACT      420
            ************************************************************

YH_G4H      GGCGCAAGATGCGGCGGATCATGACGGTGCCCTTCTTCACCAACAAGGTGGTGGCCCAGA      480
JG_G4H      GGCGCAAGATGCGGCGGATCATGACGGTGCCCTTCTTCACCAACAAGGTGGTGGCCCAGA      480
            ************************************************************

YH_G4H      ACCGCGCGGGTTGGGAGGAGGAGGCGAGGCTGGTGGTGGAGGACGTCCGCCGCGACCCCA      540
JG_G4H      ACCGCGCGGGTTGGGAGGAGGAGGCGAGGCTGGTGGTGGAGGACGTCCGCCGCGACCCCA      540
            ************************************************************

YH_G4H      CCGCGGCGACCTCCGGCGTGGTGATCCGGCGAAGGTTGCAGCTGATGATGTACAACGACA      600
JG_G4H      CCGCGGCGACCTCCGGCGTGGTGATCCGGCGAAGGTTGCAGCTGATGATGTACAACGACA      600
            ************************************************************

YH_G4H      TGTTCCGCATCATGTTCGACCGCCGTTTCGACAGCGTGGACGACCCGCTCTTCAACAAGC      660
JG_G4H      TGTTCCGCATCATGTTCGACCGCCGTTTCGACAGCGTGGACGACCCGCTCTTCAACAAGC      660
            ************************************************************

YH_G4H      TCAAGGCCTTCAACGCGGAGCGCAGCCGCCTCTCGCAGAGCTTCGAGTACAACTACGGTG      720
JG_G4H      TCAAGGCCTTCAACGCGGAGCGCAGCCGCCTCTCGCAGAGCTTCGAGTACAACTACGGTG      720
            ************************************************************

YH_G4H      ACTTCATCCCCGTCCTCCGCCCCTTCCTCCGCCGCTACCTCGCACGCTGCCACCAGCTCA      780
JG_G4H      ACTTCATCCCCGTCCTCCGCCCCTTCCTCCGCCGCTACCTCGCACGCTGCCACCAGCTCA      780
            ************************************************************

YH_G4H      AGTCCCAGCGCATGAAGCTCTTCGAGGACCACTTCGTCCAGGAACGCAAGAGAGTGATGG      840
JG_G4H      AGTCCCAGCGCATGAAGCTCTTCGAGGACCACTTCGTCCAGGAACGCAAGAGAGTGATGG      840
            ************************************************************

YH_G4H      AGCAGACTGGTGAGATCCGGTGCGCCATGGACCACATCCTCGAGGCCGAGAGGAAGGGCG      900
JG_G4H      AGCAGACTGGTGAGATCCGGTGCGCCATGGACCACATCCTCGAGGCCGAGAGGAAGGGCG      900
            ************************************************************

YH_G4H      AGATCAACCACGACAACGTCCTCTACATCGTCGAGAACATCAACGTTGCTGCTATCGAGA      960
JG_G4H      AGATCAACCACGACAACGTCCTCTACATCGTCGAGAACATCAACGTTGCTGCTATCGAGA      960
            ************************************************************

YH_G4H      CGACGCTGTGGTCGATCGAATGGGGAATCGCGGAGCTGGTGAACCACCCGAGCATCCAGT      1020
JG_G4H      CGACGCTGTGGTCGATCGAATGGGGAATCGCGGAGCTGGTGAACCACCCGAGCATCCAGT      1020
```

```
                     ************************************************************

YH_G4H               CGAAGGTGCGGGAGGAGATGGCGTCGGTGCTGGGCGGCGCGGCGGTGACGGAGCCGGACC            1080
JG_G4H               CGAAGGTGCGGGAGGAGATGGCGTCGGTGCTGGGCGGCGCGGCGGTGACGGAGCCGGACC            1080
                     ************************************************************

YH_G4H               TGGAGCGGCTGCCGTACCTGCAGGCGGTGGTGAAGGAGACGCTGCGGTTGCGCATGGCGA            1140
JG_G4H               TGGAGCGGCTGCCGTACCTGCAGGCGGTGGTGAAGGAGACGCTGCGGTTGCGCATGGCGA            1140
                     ************************************************************

YH_G4H               TCCCGCTGCTGGTGCCGCACATGAACCTCGCCGACGGCAAGCTCGCCGGCTACGACATCC            1200
JG_G4H               TCCCGCTGCTGGTGCCGCACATGAACCTCGCCGACGGCAAGCTCGCCGGCTACGACATCC            1200
                     ************************************************************

YH_G4H               CCGCCGAGTCCAAGATCCTGGTGAACGCGTGGTTCCTCGCCAACGACCCCAAGCGGTGGG            1260
JG_G4H               CCGCCGAGTCCAAGATCCTGGTGAACGCGTGGTTCCTCGCCAACGACCCCAAGCGGTGGG            1260
                     ************************************************************

YH_G4H               TGCGCCCCGACGAGTTTAGGCCGGAGAGGTTCCTGGAGGAGGAGAAGGCCGTGGAGGCGC            1320
JG_G4H               TGCGCCCCGACGAGTTTAGGCCGGAGAGGTTCCTGGAGGAGGAGAAGGCCGTGGAGGCGC            1320
                     ************************************************************

YH_G4H               ACGGCAACGACTTCCGCTTCGTGCCCTTCGGCGTCGGCCGCCGCAGCTGCCCCGGGATCA            1380
JG_G4H               ACGGCAACGACTTCCGCTTCGTGCCCTTCGGCGTCGGCCGCCGCAGCTGCCCCGGGATCA            1380
                     ************************************************************

YH_G4H               TCCTCGCGCTGCCCATCATCGGGATCACGCTCGGCCGCCTCGTCCAGAGCTTCGACCTGC            1440
JG_G4H               TCCTCGCGCTGCCCATCATCGGGATCACGCTCGGCCGCCTCGTCCAGAGCTTCGACCTGC            1440
                     ************************************************************

YH_G4H               TGCCGCCGCCCGGGATGGACAAGGTGGACACCACCGAGAAGCCCGGCCAGTTCAGCAACC            1500
JG_G4H               TGCCGCCGCCCGGGATGGACAAGGTGGACACCACCGAGAAGCCCGGCCAGTTCAGCAACC            1500
                     ************************************************************

YH_G4H               AGATCCTCAAGCACGCCACCGTCGTCTGCAAGCCCATCGACGCCTAGGTCGATCGATCGA            1560
JG_G4H               AGATCCTCAAGCACGCCACCGTCGTCTGCAAGCCCATCGACGCCTAGGTCGATCGATCGA            1560
                     ************************************************************

YH_G4H               CCTACTCAAATCAATCATGTGCTGCGTTATACTGTTGCACTAAAAAAAGGTTGTATTTTT            1620
JG_G4H               CCTACTCAAATCAATCATGTGCTGCGTTATCCTATTGCACTAAAAAAAGGTTGTATTTTT            1620
                     ****************************** ** **************************
```
