# Supplementary material for: Comparison of leaf transcriptome in response to Rhizoctonia solani infection between resistant and susceptible rice cultivars
Source: BMC Genomics. 2020 Mar 19;21:245. doi: 10.1186/s12864-020-6645-6 (PMC7081601; doi:10.1186/s12864-020-6645-6)
Supplement: Supplementary file 7 — Additional file 7: Supplementary Alignment file 3 Sequence alignment of EIN2 isoform 1 gene between JG and YH. * indicates identical position. [file 12864_2020_6645_MOESM7_ESM.pdf]

YH AGCTTGGATTCCTCTGGTTTTTTTTTTCCTTTTTGCGTACGAATCTCTTGTATATCCCTC 60
JG AGCTTGGATTCCTCTGGTTTTTTTTTTCCTTTTTGCGTACGAATCTCTTGTATATCCCTC 60
****

YH TCCTGTATGTTGCGAGAAATCAATCTAGTCCGAATGGTGTGCTCCTGCGTGCTGGAGTAG 120
JG TCCTGTATGTTGCGAGAAATCAATCTAGTCCGAATGGTGTGCTCCTGCGTGCTGGAGTAG 120
****

YH TCTAGGTATACCAGTTTTGTTCTCCAAAGCATTTGGGATTGGGTTGAATGGAACATCTAT 180
JG TCTAGGTATACCAGTTTTGTTCTCCAAAGCATTTGGGATTGGGTTGAATGGAACATCTAT 180
****

YH GGTCCACGCTAGGTCCGCCCCTGGCTGTGATGGATGGAGCTTAGAGCAGGGGCAACCTAG 240
JG GGTCCACGCTAGGTCCGCCCCTGGCTGTGATGGATGGAGCTTAGAGCAGGGGCAACCTAG 240
****

YH ATCAGGGGTGTCTCCCTGGGGGGTTTTCATGGTGCTGTGGATGTTCACTAGCTTTGGAGG 300
JG ATCAGGGGTGTCTCCCTGGGGGGTTTTCATGGTGCTGTGGATGTTCACTAGCTTTGGAGG 300
****

YH GTTTGATTAGGGCCTCTTGGGTCATAACAGGAGGGTTTTAAGGGACCTGGATTTGGTAAA 360
JG GTTTGATTAGGGCCTCTTGGGTCATAACAGGAGGGTTTTAAGGGACCTGGATTTGGTAAA 360
****

YH GCATTTTTCAGGCATGTGATGCTTCTTGGGGAGGATTTCATGGTGTTGTGGATGTTCACC 420
JG GCATTTTTCAGGCATGTGATGCTTCTTGGGGAGGATTTCATGGTGTTGTGGATGTTCACC 420
****

YH AGCTTTGGAGGGTTTGATTAGGGCTGCTTGGGTCACAACAGGAGGGCTTTAGGGATCTGA 480
JG AGCTTTGGAGGGTTTGATTAGGGCTGCTTGGGTCACAACAGGAGGGCTTTAGGGATCTGA 480
****

YH ATTTGGTAGTCATTTCAGGCATGTAATGCTTCTTGGGGAGGATTTCATGGTGTTGTGGAT 540
JG ATTTGGTAGTCATTTCAGGCATGTAATGCTTCTTGGGGAGGATTTCATGGTGTTGTGGAT 540
****

YH GGATGTTCCCCAGGTTTGGATGGTTAGACTTCTTCGATCACCACAAGAGCTTTGAAGAGA 600
JG GGATGTTCCCCAGGTTTGGATGGTTAGACTTCTTCGATCACCACAAGAGCTTTGAAGAGA 600
****

YH CCTGAGTGGATAAGCATTTGATCCTTCTTGGAGGTGTTTCATGGTGTTCTAGATGTTCAC 660
JG CCTGAGTGGATAAGCATTTGATCCTTCTTGGAGGTGTTTCATGGTGTTCTAGATGTTCAC 660
****

YH CAGCTTCGGAGGGTTTGATTAGACTGCTTGAGTTAACAGTGTTCAAGGGACTGAATTTGA 720
JG CAGCTTCGGAGGGTTTGATTAGACTGCTTGAGTTAACAGTGTTCAAGGGACTGAATTTGA 720
****

YH TAAGCATGTCGGGCATTTGATCCAGTGGTGTGGATTCGTATCCATCTTTTGTTGTTATAA 780
JG TAAGCATGTCGGGCATTTGATCCAGTGGTGTGGATTCGTATCCATCTTTTGTTGTTATAA 780
****

YH AATTTGCTGCCACAAAAAAATGGAAGGTGTGCGCGGTATAGAATCTCTGGCTACTGGAGAT 840
JG AATTTGCTGCCACAAAAAAATGGAAGGTGTGCGCGGTATAGAATCTCTGGCTACTGGAGAT 840
****

YH GGTCGGCATCATCTTTCCCGTACCCTTGGACCGGTGCTCCTGATCTCGATGGGGTATATT 900
JG GGTCGGCATCATCTTTCCCGTACCCTTGGACCGGTGCTCCTGATCTCGATGGGGTATATT 900
****

YH GACCTTGGGAAGTGGGTGGCAACGATAGATGCCGGGTCTCGGTTTGGCTATGATCTCGTA 960
JG GACCTTGGGAAGTGGGTGGCAACGATAGATGCCGGGTCTCGGTTTGGCTATGATCTCGTA 960
****

YH ATACTGGTGTTGCTTTTCAACTTGTCGGCCATTCTGTGCCAGTATCTGTCGATGTGTATC 1020
JG ATACTGGTGTTGCTTTTCAACTTGTCGGCCATTCTGTGCCAGTATCTGTCGATGTGTATC 1020

```
            ************************************************************

YH          GGCATGGTCACTGGGAAAAATCTTGCGGAGATTTGCCGCGAGGAGTACAGTCCATCAATA      1080
JG          GGCATGGTCACTGGGAAAAATCTTGCGGAGATTTGCCGCGAGGAGTACAGTCCATCAATA      1080
            ************************************************************

YH          TGTGTCATCCTTGGTATTCAGGCAGGATTGTCCTTGCTAACCGCGGAACTAACCATGCTT      1140
JG          TGTGTCATCCTTGGTATTCAGGCAGGATTGTCCTTGCTAACCGCGGAACTAACCATGCTT      1140
            ************************************************************

YH          TCAGGCATATCAGTTGGATTCAACCTGGTCTTTGAATATGATGATCCTATCGCAGGCTTA      1200
JG          TCAGGCATATCAGTTGGATTCAACCTGGTCTTTGAATATGATGATCCTATCGCAGGCTTA      1200
            ************************************************************

YH          TATTTTGCTAGTGTTGTGGTCAATTTGCTACCTTACACTATGTCTTATCTGGGCAAACGG      1260
JG          TATTTTGCTAGTGTTGTGGTCAATTTGCTACCTTACACTATGTCTTATCTGGGCAAACGG      1260
            ************************************************************

YH          ATGGCTGGGACATTGAATGCATGCGTAGCAGGCTTTGCACTTCTTTGTTTTGTGCTTGGT      1320
JG          ATGGCTGGGACATTGAATGCATGCGTAGCAGGCTTTGCACTTCTTTGTTTTGTGCTTGGT      1320
            ************************************************************

YH          TTATTAGTCAGTCAACCAAAAATTCCAGTTGATATGAATGCAATGTTCCCCAAGTTGAGT      1380
JG          TTATTAGTCAGTCAACCAAAAATTCCAGTTGATATGAATGCAATGTTCCCCAAGTTGAGT      1380
            ************************************************************

YH          GGTGAAAGTGCTTATTCCTTGATGGCGCTTCTTGGCGGAAATGTAATAGCGCACAATTTT      1440
JG          GGTGAAAGTGCTTATTCCTTGATGGCGCTTCTTGGCGGAAATGTAATAGCGCACAATTTT      1440
            ************************************************************

YH          TATGTTCATTCATCAGTTGTACAGGGCCAAAGACAATCTACAACTCTTTCCCTTGGTGCT      1500
JG          TATGTTCATTCATCAGTTGTACAGGGCCAAAGACAATCTACAACTCTTTCCCTTGGTGCT      1500
            ************************************************************

YH          CTGTTCCACGATCACCTGTTCTCAATATTGTTTATTTTCACTGGGGTTTTCCTTGTGAAT      1560
JG          CTGTTCCACGATCACCTGTTCTCAATATTGTTTATTTTCACTGGGGTTTTCCTTGTGAAT      1560
            ************************************************************

YH          TATGTCCTGATGGGCTCAGCAGCAGTTGAATCCAATAATACTCTGGTTACTTTTCAAGAT      1620
JG          TATGTCCTGATGGGCTCAGCAGCAGTTGAATCCAATAATACTCTGGTTACTTTTCAAGAT      1620
            ************************************************************

YH          TCTGTAGATTTAATGAACCAGATGTTCATGAATCCGATGGCACCAATTGTTTTTTTAGTG      1680
JG          TCTGTAGATTTAATGAACCAGATGTTCATGAATCCGATGGCACCAATTGTTTTTTTAGTG      1680
            ************************************************************

YH          ATCCTTATCTTTTCGAGTCATGTCATCTCATTGACATCTATTATTGGCAGCCACGCAATT      1740
JG          ATCCTTATCTTTTCGAGTCATGTCATCTCATTGACATCTATTATTGGCAGCCACGCAATT      1740
            ************************************************************

YH          TTGAAGAATTTCTTTGGTGTAAACTTGCCTCATTCTGCTCATCATCTGCTACTAAAGGCC      1800
JG          TTGAAGAATTTCTTTGGTGTAAACTTGCCTCATTCTGCTCATCATCTGCTACTAAAGGCC      1800
            ************************************************************

YH          GTTGCCATGGTTCCTACTATGTACTATGCAAAGGTTGCAGGTTCTGAAGGGATATATCAG      1860
JG          GTTGCCATGGTTCCTACTATGTACTATGCAAAGGTTGCAGGTTCTGAAGGGATATATCAG      1860
            ************************************************************

YH          TTACTCATTATCTGCCCAGTTATCCAAGCTATGTTCCTTCCTTCATCTGTTATTCCTGTT      1920
JG          TTACTCATTATCTGCCCAGTTATCCAAGCTATGTTCCTTCCTTCATCTGTTATTCCTGTT      1920
            ************************************************************

YH          TTCCGTGTTTCCTCATCAAGAGTTATAATGAGCAGATATAAAATATCTTTGTACGTTGAA      1980
JG          TTCCGTGTTTCCTCATCAAGAGTTATAATGAGCAGATATAAAATATCTTTGTACGTTGAA      1980
            ************************************************************

YH          ATATTGTCCATCCTAGCATTTCTTCTTTTGCTGTTCACAAATATCATTTTTGCTGCGGAA      2040
JG          ATATTGTCCATCCTAGCATTTCTTCTTTTGCTGTTCACAAATATCATTTTTGCTGCGGAA      2040
            ************************************************************
```

```
YH      ATCCTGTTTGGTGATAGTACCTGGACAAACAACTTGAAAGGGAACGCTGGAAGCCCTGTT    2100
JG      ATCCTGTTTGGTGATAGTACCTGGACAAACAACTTGAAAGGGAACGCTGGAAGCCCTGTT    2100
        ************************************************************

YH      GTACTTCCGCATGCCATTGTAGTTCTAATTTCTTGTGCATCAATTACTTTTACGCTGTTC    2160
JG      GTACTTCCGCATGCCATTGTAGTTCTAATTTCTTGTGCATCAATTACTTTTACGCTGTTC    2160
        ************************************************************

YH      CTGGCTGTCACTCCACTGAAGTCAGCAAGTAATGAACCTGAAACTCAGGAGCTATCTGAG    2220
JG      CTGGCTGTCACTCCACTGAAGTCAGCAAGTAATGAACCTGAAACTCAGGAGCTATCTGAG    2220
        ************************************************************

YH      CACTCTCAGAGAGAAGATCCAGATACTACTTATCAAAGAGAAGCAAGTAATGAACCTGAA    2280
JG      CACTCTCAGAGAGAAGATCCAGATACTACTTATCAAAGAGAAGCAAGTAATGAACCTGAA    2280
        ************************************************************

YH      ACTCAGGAGCTATCTGAGCACTCTCAGAGAGAAGATCCAGATACTACTTATCAAATAGAA    2340
JG      ACTCAGGAGCTATCTGAGCACTCTCAGAGAGAAGATCCAGATACTACTTATCAAATAGAA    2340
        ************************************************************

YH      GTAAGTAATGAACGTGAAACTCAGCAGCTATCTGAGCACTCTCAGATAGAAGATCCAGAT    2400
JG      GTAAGTAATGAACGTGAAACTCAGCAGCTATCTGAGCACTCTCAGATAGAAGATCCAGAT    2400
        ************************************************************

YH      ACTTTTTATCATAGAGAGGAGCTTTCTCTGGTTGAACAGAAAGAAGATCATACGACTTCT    2460
JG      ACTTTTTATCATAGAGAGGAGCTTTCTCTGGTTGAACAGAAAGAAGATCATACGACTTCT    2460
        ************************************************************

YH      ACTATTAATGCTATTCCCAGGATTTCATCAGAAAGTTATCAAACATCAGCTTTGGAGCAT    2520
JG      ACTATTAATGCTATTCCCAGGATTTCATCAGAAAGTTATCAAACATCAGCTTTGGAGCAT    2520
        ************************************************************

YH      AATGACTTTCCTGACATCACTGTGGAGTCTGGTCATGGCACTCAGCAGCTTACTGCTTTT    2580
JG      AATGACTTTCCTGACATCACTGTGGAGTCTGGTCATGGCACTCAGCAGCTTACTGCTTTT    2580
        ************************************************************

YH      GTGCCAATTATTCCGGAGGTCTCATCGTCTATCAAACATAAGGAACCAAAATCAGTAGTT    2640
JG      GTGCCAATTATTCCGGAGGTCTCATCGTCTATCAAACATAAGGAACCAAAATCAGTAGTT    2640
        ************************************************************

YH      ATTGACCAGACGGAACCAGTGCCAAAGGTTTGTACTGCCACAGTAGTAGAACATAACACT    2700
JG      ATTGACCAGACGGAACCAGTGCCAAAGGTTTGTACTGCCACAGTAGTAGAACATAACACT    2700
        ************************************************************

YH      GCTGAGAACATCAAAATGAAGAGTACAACTTCAAAGCATGTCCAAGAAGAAGCAGGAGCT    2760
JG      GCTGAGAACATCAAAATGAAGAGTACAACTTCAAAGCATGTCCAAGAAGAAGCAGGAGCT    2760
        ************************************************************

YH      AGCATGGACTATGATACTGAGGCTTCTTATAATGCGGAAGTCAGCAAGTCTTCTGGAAAC    2820
JG      AGCATGGACTATGATACTGAGGCTTCTTATAATGCGGAAGTCAGCAAGTCTTCTGGAAAC    2820
        ************************************************************

YH      AAGGCACCTCCAATTTCTGATGACCCAACATCTCTTACTTTGAGCAAGGGGAGAGACTCT    2880
JG      AAGGCACCTCCAATTTCTGATGACCCAACATCTCTTACTTTGAGCAAGGGGAGAGACTCT    2880
        ************************************************************

YH      GATGCTGGTTATCGTGGCAGTAACCTCTCAAGACTGCCTGGTTTGGGTCGTGCAGCAAGG    2940
JG      GATGCTGGTTATCGTGGCAGTAACCTCTCAAGACTGCCTGGTTTGGGTCGTGCAGCAAGG    2940
        ************************************************************

YH      AGGCAATTAGCAGCGATTCTTGATGAGTTCTGGGGACATCTCTTTGATTATCATGGTAAG    3000
JG      AGGCAATTAGCAGCGATTCTTGATGAGTTCTGGGGACATCTCTTTGATTATCATGGTAAG    3000
        ************************************************************

YH      CTAACGCAAGAAGCTAATGCAGGAAGGTTCAACTTTCTGCTAGGACCATACCCGAAAGCA    3060
JG      CTAACGCAAGAAGCTAATGCAGGAAGGTTCAACTTTCTGCTAGGACCATACCCGAAAGCA    3060
        ************************************************************
```

```
YH      GTTAGAAGTGATAACCAAGCCATCGAAGCTTCTAGGAGCCCCTTGATGAGAGATGCAATA    3120
JG      GTTAGAAGTGATAACCAAGCCATCGAAGCTTCTAGGAGCCCCTTGATGAGAGATGCAATA    3120
        ************************************************************

YH      CGAGGATCAGCTACCATACAGAAATCATGGGACTCACGTGCTAAGGAAGTCTCTAGTCCA    3180
JG      CGAGGATCAGCTACCATACAGAAATCATGGGACTCACGTGCTAAGGAAGTCTCTAGTCCA    3180
        ************************************************************

YH      GGCTTTAATTTTGGGCTTCAGATGGGTCGCATTGGATCATCAAACTGGTCTGAGAGCATG    3240
JG      GGCTTTAATTTTGGGCTTCAGATGGGTCGCATTGGATCATCAAACTGGTCTGAGAGCATG    3240
        ************************************************************

YH      CGTTTATCTAATGCTGACATCCCAAGGCCAACTAGCACCTTGTTTGAACAAAATACTCAG    3300
JG      CGTTTATCTAATGCTGACATCCCAAGGCCAACTAGCACCTTGTTTGAACAAAATACTCAG    3300
        ************************************************************

YH      TTTTATTCAAATTATAATGTCCCATCTTACCCTGACAATCAGTTCTATCAACCTGCTACC    3360
JG      TTTTATTCAAATTATAATGTCCCATCTTACCCTGACAATCAGTTCTATCAACCTGCTACC    3360
        ************************************************************

YH      ATTCATGGCTATCACCTGGCAACCTCTTTGAAAAGTATGAATGCAAGTCACAGCACGCAC    3420
JG      ATTCATGGCTATCACCTGGCAACCTCTTTGAAAAGTATGAATGCAAGTCACAGCACGCAC    3420
        ************************************************************

YH      TCCAGCATTTCACTAGATCCACGGCGACTTCCTAGATCATCTGAATCTGCTGGTTCTAAC    3480
JG      TCCAGCATTTCACTAGATCCACGGCGACTTCCTAGATCATCTGAATCTGCTGGTTCTAAC    3480
        ************************************************************

YH      TACGCAGATTCTGCAAGGTATGCTCGTAACCAAGATGTAATTGGTTCACAGGGAACCGCT    3540
JG      TACGCAGATTCTGCAAGGTATGCTCGTAACCAAGATGTAATTGGTTCACAGGGAACCGCT    3540
        ************************************************************

YH      TCGCAAAACACAACAATGAGCTGTTTAGATACAATGACAGTGGAGAGAGCTTTTTACAAT    3600
JG      TCGCAAAACACAACAATGAGCTGTTTAGATACAATGACAGTGGAGAGAGCTTTTTACAAT    3600
        ************************************************************

YH      CCTGCCTCCGTTAATGAGATTGAAGGGGTTGGTTCATCTGCTTACTCAAAGAAGTACCAT    3660
JG      CCTGCCTCCGTTAATGAGATTGAAGGGGTTGGTTCATCTGCTTACTCAAAGAAGTACCAT    3660
        ************************************************************

YH      AGTTCACCTGACATATCTGCACTAATTGCTGCAAGTAGGAATTATTTGCCAAATGAAGTA    3720
JG      AGTTCACCTGACATATCTGCACTAATTGCTGCAAGTAGGAATTATTTGCCAAATGAAGTA    3720
        ************************************************************

YH      AATTTGGGAGGTGCTGCTGGAAGCAGTTCATACTTCAGTAATTTGGCATGTGAAAGATCA    3780
JG      AATTTGGGAGGTGCTGCTGGAAGCAGTTCATACTTCAGTAATTTGGCATGTGAAAGATCA    3780
        ************************************************************

YH      CAATATGTGAACTTGGGATCCAGTTCCACAGCTCAATTTGCACTTAGCAAGCACTCACAA    3840
JG      CAATATGTGAACTTGGGATCCAGTTCCACAGCTCAATTTGCACTTAGCAAGCACTCACAA    3840
        ************************************************************

YH      CCTAATTTCCATAGAGACACATCATCTATGCAGTCAAGTGTGAACCCAAGTACTGAATCC    3900
JG      CCTAATTTCCATAGAGACACATCATCTATGCAGTCAAGTGTGAACCCAAGTACTGAATCC    3900
        ************************************************************

YH      ATTTGGGCCCAGCAGCCGTTTGAACAATTACTCGGTGTATCAAGAGCAGAGTTGAATAAG    3960
JG      ATTTGGGCCCAGCAGCCGTTTGAACAATTACTCGGTGTATCAAGAGCAGAGTTGAATAAG    3960
        ************************************************************

YH      GGCGAGGGTAACACCGACCAGAGATCAAGTGGTGTCACCAAACACGATTTCACTAACAAA    4020
JG      GGCGAGGGTAACACCGACCAGAGATCAAGTGGTGTCACCAAACACGATTTCACTAACAAA    4020
        ************************************************************

YH      GAATATGAGGTGAAACTTCTTCAATCACTCAGATTTTGCATCATGAAGCTCTTGAAACTG    4080
JG      GAATATGAGGTGAAACTTCTTCAATCACTCAGATTTTGCATCATGAAGCTCTTGAAACTG    4080
        ************************************************************

YH      GAAGGATCAGGATGGCTCTTTGAGCAAAATGGTGGCTGTGATGAAAAATTAATTGATCAA    4140
```

```
JG      GAAGGATCAGGATGGCTCTTTGAGCAAAATGGTGGCTGTGATGAAAAATTAATTGATCAA    4140
        ************************************************************

YH      GTTGCTGTAGCTGAGAGAGTTTCACAACATACCACTGAAAATCAGTTATCTGCTGATCTC    4200
JG      GTTGCTGTAGCTGAGAGAGTTTCACAACATACCACTGAAAATCAGTTATCTGCTGATCTC    4200
        ************************************************************

YH      CAGCTCCATAGTTCTGATGAAGACTTGCAGCCACTGCAAAGGAATGATAACAGGGATGCC    4260
JG      CAGCTCCATAGTTCTGATGAAGACTTGCAGCCACTGCAAAGGAATGATAACAGGGATGCC    4260
        ************************************************************

YH      AATTGCATGAGCCTACTGCCCAAGTGTGGAGATGATTGTGTTTGGCAGGCCCCCCTGATT    4320
JG      AATTGCATGAGCCTACTGCCCAAGTGTGGAGATGATTGTGTTTGGCAGGCCCCCCTGATT    4320
        ************************************************************

YH      GTTAGTTTTGGTGTCTGGTGCATCCGCCAGATTCTGAACCTGTGCCTTGTCGAAAGTAGG    4380
JG      GTTAGTTTTGGTGTCTGGTGCATCCGCCAGATTCTGAACCTGTGCCTTGTCGAAAGTAGG    4380
        ************************************************************

YH      CCAGAACTTTGGGGCAAGTATACATATGTTCTTAATCGTCTCCAGGGAATACTTGATCCT    4440
JG      CCAGAACTTTGGGGCAAGTATACATATGTTCTTAATCGTCTCCAGGGAATACTTGATCCT    4440
        ************************************************************

YH      GCATTTTCCAAGCCTCGGAAACCCATGAAAGGATGCGTATGCCTTCAAAAAGTTGCCAAG    4500
JG      GCATTTTCCAAGCCTCGGAAACCCATGAAAGGATGCGTATGCCTTCAAAAAGTTGCCAAG    4500
        ************************************************************

YH      CCCATCTCTGGTACTTTCACCACTGCTGGTATGATCTTGGAGATGATTAAAGACGTGGAA    4560
JG      CCCATCTCTGGTACTTTCACCACTGCTGGTATGATCTTGGAGATGATTAAAGACGTGGAA    4560
        ************************************************************

YH      CAAGCCATTTCTAGCCGCAAGGGTCGAAGCGGCACAGCAGCAGGAGACGTTGCTTTTCCC    4620
JG      CAAGCCATTTCTAGCCGCAAGGGTCGAAGCGGCACAGCAGCAGGAGACGTTGCTTTTCCC    4620
        ************************************************************

YH      AAAGGGAAGGAGAACCTAGCTTCTGTCCTTAAGCGATACAAGCGTAGGCTCTCGAACAAG    4680
JG      AAAGGGAAGGAGAACCTAGCTTCTGTCCTTAAGCGATACAAGCGTAGGCTCTCGAACAAG    4680
        ************************************************************

YH      ACATCTGCAGGACAATAGCGTGGCAGCGAGCTTTCTTTTGTTTCTTGTTTGTATAGGGTT    4740
JG      ACATCTGCAGGACAATAGCGTGGCAGCGAGCTTTCTTTTGTTTCTTGTTTGTATAGGGTT    4740
        ************************************************************

YH      CTTGGGGCTGCTCCACAAAGTTCTGTTTTTTTGTGCTCCTCAAACCTTGGGTTTTTTCGA    4800
JG      CTTGGGGCTGCTCCACAAAGTTCTGTTTTTTTGTGCTCCTCAAACCTTGGGTTTTTTCGA    4800
        ************************************************************

YH      TGCACACGATCTCCAGAGTGCCTGAGAGCTTCTTGATCTTTGGTCATTTTTGCACATGTT    4860
JG      TGCACACGATCTCCAGAGTGCCTGAGAGCTTCTTGATCTTTGGTCATTTTTGCACATGTT    4860
        ************************************************************

YH      GTTTATGAAGTGGCCAAGGGTGAATGGTATACCTTGTTTATTCATCTTATCAGCGAGATC    4920
JG      GTTTATGAAGTGGCCAAGGGTGAATGGTATACCTTGTTTATTCATCTTATCAGCGAGATC    4920
        ************************************************************

YH      TCAACAGTAGATGATATTTGCTGGAGCAGCAACATTGTAAAGTTCTTTTTCCAGATGAAC    4980
JG      TCAACAGTAGATGATATTTGCTGGAGCAGCAACATTGTAAAGTTCTTTTTCCAGATGAAC    4980
        ************************************************************

YH      GTTCTGAAGTCCGCTGGCTTGGCTTTCTGAAAAAAAAAAAAAAAAA    5026
JG      GTTCTGAAGTCCGCTGGCTTGGCTTTCTGAAAAAAAAAAAAAAAAA    5026
        **********************************************
```
