# Supplementary material for: Comparison of leaf transcriptome in response to Rhizoctonia solani infection between resistant and susceptible rice cultivars
Source: BMC Genomics. 2020 Mar 19;21:245. doi: 10.1186/s12864-020-6645-6 (PMC7081601; doi:10.1186/s12864-020-6645-6)
Supplement: Supplementary file 8 — Additional file 8: Supplementary Alignment file 4 Sequence alignment of EIN2 isoform 2 gene between JG and YH. * indicates identical position. [file 12864_2020_6645_MOESM8_ESM.pdf]

```
JG     CTAGCTTCTTCTCCTCCTCCTCCTCCCCCTTCTCCCCGTAGATAAGGAGGACCCGGGGAA    60
YH     CTAGCTTCTTCTCCTCCTCCTCCTCCCCCTTCTCCCCGTAGATAAGGAGGACCCGGGGAA    60
       ************************************************************

JG     AAAGGGGAGGCAAAAAGCTGCGATTTTCCGCTAATTAACCCCGGGGATTAGCTGCCCAAG    120
YH     AAAGGGGAGGCAAAAAGCTGCGATTTTCCGCTAATTAACCCCGGGGATTAGCTGCCCAAG    120
       ************************************************************

JG     AACAGGAGGAGATTTCCATGATTGGTGGTGGAGTTCATCGGCAAGGAATTCAGCAGAAGA    180
YH     AACAGGAGGAGATTTCCATGATTGGTGGTGGAGTTCATCGGCAAGGAATTCAGCAGAAGA    180
       ************************************************************

JG     GATCAGATTCTGTACACAGTTCGTCTTCTTGCTCGGTGACTAAGCTGGGTTAGGAGAGGA    240
YH     GATCAGATTCTGTACACAGTTCGTCTTCTTGCTCGGTGACTAAGCTGGGTTAGGAGAGGA    240
       ************************************************************

JG     GGAAAAGAAAATCTTTTTTTTTTTTTTTGCGGCGCCATGGATGGGCAGCAGCTACGTAGC    300
YH     GGAAAAGAAAATCTTTTTTTTTTTTTTTGCGGCGCCATGGATGGGCAGCAGCTACGTAGC    300
       ************************************************************

JG     TCGGAATCTCCGGCGAGCGGCGGCGGCGGAGTCACCGGCGGCGGCGCGCCACATCTGTTC    360
YH     TCGGAATCTCCGGCGAGCGGCGGCGGCGGAGTCACCGGCGGCGGCGCGCCACATCTGTTC    360
       ************************************************************

JG     CACGCGCTCGGGCCGGCGCTGCTGATCTCGATTGGGTACATTGACCTCGGGAAATGGGTG    420
YH     CACGCGCTCGGGCCGGCGCTGCTGATCTCGATTGGGTACATTGACCTCGGGAAATGGGTG    420
       ************************************************************

JG     GCCGCGGTGGAGGCAGGGTCACGGTTCGGCCTTGACCTCGTGCTGCTGGCCCTCCTCTTC    480
YH     GCCGCGGTGGAGGCAGGGTCACGGTTCGGCCTTGACCTCGTGCTGCTGGCCCTCCTCTTC    480
       ************************************************************

JG     AACTTCATGGCCATCCTGTGCCAGTATCTCGCGGCTTGCATTGGCACGGTCACCGGGAGG    540
YH     AACTTCATGGCCATCCTGTGCCAGTATCTCGCGGCTTGCATTGGCACGGTCACCGGGAGG    540
       ************************************************************

JG     AGCCTCGCCGAGATCTGCCACCAAGAATACAGCAGGCCAACATGCATCTTTCTGGGTGTT    600
YH     AGCCTCGCCGAGATCTGCCACCAAGAATACAGCAGGCCAACATGCATCTTTCTGGGTGTT    600
       ************************************************************

JG     CAAGCAGGATTGTCCTTGTTGACATCAGAATTGACGATGATTTTTGGGATAGCACTTGGA    660
YH     CAAGCAGGATTGTCCTTGTTGACATCAGAATTGACGATGATTTTTGGGATAGCACTTGGA    660
       ************************************************************

JG     TTCAATCTTCTATTTGAATATGATGATCTCATCACTGGGATATGTTTTGCAACCGTTGTT    720
YH     TTCAATCTTCTATTTGAATATGATGATCTCATCACTGGGATATGTTTTGCAACCGTTGTT    720
       ************************************************************

JG     CCTAATCTGCTACCATATGCTATATCACACCTGGGAAAGAAGATGGTGGGGACATTAAAT    780
YH     CCTAATCTGCTACCATATGCTATATCACACCTGGGAAAGAAGATGGTGGGGACATTAAAT    780
       ************************************************************

JG     GCTTGCATTGCAGGCTTTGCGCTTCTTTGCTACGTTCTTGGTTTATTGGTCAGCCAACCA    840
YH     GCTTGCATTGCAGGCTTTGCGCTTCTTTGCTACGTTCTTGGTTTATTGGTCAGCCAACCA    840
       ************************************************************

JG     CAAATTCCTCTGACAACGAATGTAATTTTCCCCAAGCTCAGTGGTGAAAGTGCTTATTCT    900
YH     CAAATTCCTCTGACAACGAATGTAATTTTCCCCAAGCTCAGTGGTGAAAGTGCTTATTCT    900
       ************************************************************

JG     CTGATGGCTCTTCTTGGTGCAAACGTAATGGCACACAACTTTTACATCCATTCATCAGTT    960
YH     CTGATGGCTCTTCTTGGTGCAAACGTAATGGCACACAACTTTTACATCCATTCATCAGTT    960
       ************************************************************

JG     GTTCAGGGTCAGAAAAGATCTGCCTTTGCTGTTGGTGCCTTATTTCATGATCACTTGTTT    1020
YH     GTTCAGGGTCAGAAAAGATCTGCCTTTGCTGTTGGTGCCTTATTTCATGATCACTTGTTT    1020
```

```
            ************************************************************

JG          TCAGTATTATTTATTTTTACTGGAATTTTTCTGGTGAATCATGTTCTAATGAACTCTGCA    1080
YH          TCAGTATTATTTATTTTTACTGGAATTTTTCTGGTGAATCATGTTCTAATGAACTCTGCA    1080
            ************************************************************

JG          GCAGCTGATTCCACTAACACCCTTCTTCTCACCTTCCAAGATGTTGTAGAACTAATGAAC    1140
YH          GCAGCTGATTCCACTAACACCCTTCTTCTCACCTTCCAAGATGTTGTAGAACTAATGAAC    1140
            ************************************************************

JG          CAGATATTTGTAAACCCTATGGCTCCAACTATATTTCTAGTGGTTCTTCTCTTCTCTAGC    1200
YH          CAGATATTTGTAAACCCTATGGCTCCAACTATATTTCTAGTGGTTCTTCTCTTCTCTAGC    1200
            ************************************************************

JG          CACATCATCTCGTTGACATCTGCTATTGGTAGCCAAGTGATTTCGCAGCATTTGTTTGGC    1260
YH          CACATCATCTCGTTGACATCTGCTATTGGTAGCCAAGTGATTTCGCAGCATTTGTTTGGC    1260
            ************************************************************

JG          ATTAATCTTCCTCTCTCTGGACATCATCTGATACTGAAGGCTTTTGCCATAGTTCCTGCT    1320
YH          ATTAATCTTCCTCTCTCTGGACATCATCTGATACTGAAGGCTTTTGCCATAGTTCCTGCT    1320
            ************************************************************

JG          CTGTACTGTGCTAAGGTTGCAGGTGCTGAAGGAATATACCAATTACTGATAATCTGCCAG    1380
YH          CTGTACTGTGCTAAGGTTGCAGGTGCTGAAGGAATATACCAATTACTGATAATCTGCCAG    1380
            ************************************************************

JG          ATTATCCAGGCCATGCTCCTTCCATCATCAGTCGTGCCACTCTTCCGTGTTGCCTCATCA    1440
YH          ATTATCCAGGCCATGCTCCTTCCATCATCAGTCGTGCCACTCTTCCGTGTTGCCTCATCA    1440
            ************************************************************

JG          AGATTGATAATGGGTGCCCACAGAGTGTCTTTGCATCTGGAGATATTAACATTTCTTGCA    1500
YH          AGATTGATAATGGGTGCCCACAGAGTGTCTTTGCATCTGGAGATATTAACATTTCTTGCA    1500
            ************************************************************

JG          TTTCTCCTCATGCTGTTTTCGAATATCATCTTTATGGCAGAAATGCTGTTTGGTGACAGT    1560
YH          TTTCTCCTCATGCTGTTTTCGAATATCATCTTTATGGCAGAAATGCTGTTTGGTGACAGT    1560
            ************************************************************

JG          GGTTGGCTGAACACTCTGAAAGGGAATACTGGAAGCCCTGTGGTGTTTCCATCTACGGTT    1620
YH          GGTTGGCTGAACACTCTGAAAGGGAATACTGGAAGCCCTGTGGTGTTTCCATCTACGGTT    1620
            ************************************************************

JG          CTCATCACGGTGGCTTGTGTCTCTGTTGCATTTTCACTCTACATGGCTGTTACACCACTG    1680
YH          CTCATCACGGTGGCTTGTGTCTCTGTTGCATTTTCACTCTACATGGCTGTTACACCACTG    1680
            ************************************************************

JG          AAATCAGGAAGCCATGAAGCTGAATTGCAGCAGGAATGGTCTGTGCCTTCTCAGAAAGAG    1740
YH          AAATCAGGAAGCCATGAAGCTGAATTGCAGCAGGAATGGTCTGTGCCTTCTCAGAAAGAG    1740
            ************************************************************

JG          CTCTTGAATACTACTCAAGACAGAGAAGAGACTTGTGCGGGCAATGTTACCTATGAGGAA    1800
YH          CTCTTGAATACTACTCAAGACAGAGAAGAGACTTGTGCGGGCAATGTTACCTATGAGGAA    1800
            ************************************************************

JG          GATCAGAGATCTGATGTTGTCCCTTCTCCTAGGATTCAGCCTGTGGATTGTCTGAAATCA    1860
YH          GATCAGAGATCTGATGTTGTCCCTTCTCCTAGGATTCAGCCTGTGGATTGTCTGAAATCA    1860
            ************************************************************

JG          GCTCTGGACTACATTGATAGTTCGGACACAGCTATAGAATCTGATCATGATTCTCAACAT    1920
YH          GCTCTGGACTACATTGATAGTTCGGACACAGCTATAGAATCTGATCATGATTCTCAACAT    1920
            ************************************************************

JG          TCCACTGCTCATACATCTACCGCTCCTGAATCCTGTCACTCTCCATCATTCATTCCTGAA    1980
YH          TCCACTGCTCATACATCTACCGCTCCTGAATCCTGTCACTCTCCATCATTCATTCCTGAA    1980
            ************************************************************

JG          GAGTCAAAATCAGTTGTTGCTGTTGACTGGCCAGAGCCTCTGGAGCCAATTTCTAATGCT    2040
YH          GAGTCAAAATCAGTTGTTGCTGTTGACTGGCCAGAGCCTCTGGAGCCAATTTCTAATGCT    2040
            ************************************************************
```

```
JG      ATTGTGGCTGAGGAAAGTACAGTAGAGAGTGTGGACTCCAAGAGCACAGGCGAAAGGGAT    2100
YH      ATTGTGGCTGAGGAAAGTACAGTAGAGAGTGTGGACTCCAAGAGCACAGGCGAAAGGGAT    2100
        ************************************************************

JG      ATTGAAGTAGAACCAGCTCTTTTGATGGACAATGATAAGGAGGCTCCAAATATTCTAGAG    2160
YH      ATTGAAGTAGAACCAGCTCTTTTGATGGACAATGATAAGGAGGCTCCAAATATTCTAGAG    2160
        ************************************************************

JG      TCTGACAACAAGCCACTTGGAGGCAATAATCCTTCCTGTGCATCGGATGATGGCCCACCA    2220
YH      TCTGACAACAAGCCACTTGGAGGCAATAATCCTTCCTGTGCATCGGATGATGGCCCACCA    2220
        ************************************************************

JG      TCTCTTACCTTCAGCAGGGGGAAAGGCTCAGATGCAGGCAATGGCAGCGGGAGTCTCTCG    2280
YH      TCTCTTACCTTCAGCAGGGGGAAAGGCTCAGATGCAGGCAATGGCAGCGGGAGTCTCTCG    2280
        ************************************************************

JG      AGGTTATCTGGTTTGGGCCGTGCAGCAAGGAGGCAACTAGCAGCCATACTTGATGAGTTC    2340
YH      AGGTTATCTGGTTTGGGCCGTGCAGCAAGGAGGCAACTAGCAGCCATACTTGATGAGTTC    2340
        ************************************************************

JG      TGGGGGCATCTCTTTGATTACCATGGGAAACTCACTCAAGAAGCTAGCTCTAAAAGGTTT    2400
YH      TGGGGGCATCTCTTTGATTACCATGGGAAACTCACTCAAGAAGCTAGCTCTAAAAGGTTT    2400
        ************************************************************

JG      GACATCTTGCTTGGGCTAGACGTAAGAACACCTAGCTCAACTGTAAGAGCAGACAGTCAA    2460
YH      GACATCTTGCTTGGGCTAGACGTAAGAACACCTAGCTCAACTGTAAGAGCAGACAGTCAA    2460
        ************************************************************

JG      GCTAATGAAATCCCGAAGAGTCCCATGGTACGAGACAATTTACAAGGGTCTGCCTTCTTG    2520
YH      GCTAATGAAATCCCGAAGAGTCCCATGGTACGAGACAATTTACAAGGGTCTGCCTTCTTG    2520
        ************************************************************

JG      GGAAGTTCAAGGGATCTGATGTCTACTAAGAATGAGATGTCGAATTTGGATCTGACATAT    2580
YH      GGAAGTTCAAGGGATCTGATGTCTACTAAGAATGAGATGTCGAATTTGGATCTGACATAT    2580
        ************************************************************

JG      GGGCTTCAGATGGGCAATAACATTGGGTCATCAGCCTGGTCTCAGGGCATGCAGTTACCA    2640
YH      GGGCTTCAGATGGGCAATAACATTGGGTCATCAGCCTGGTCTCAGGGCATGCAGTTACCA    2640
        ************************************************************

JG      AGTACCCAACTGCAGAGTTCAAGCAACAGCTTACTCGATCAAGGTGCAAGATTAAATTCA    2700
YH      AGTACCCAACTGCAGAGTTCAAGCAACAGCTTACTCGATCAAGGTGCAAGATTAAATTCA    2700
        ************************************************************

JG      AATTTTAGCACGCCATCATACGCAGACAACAACCAATTCTACCAACCTGCAACGATTCAT    2760
YH      AATTTTAGCACGCCATCATACGCAGACAACAACCAATTCTACCAACCTGCAACGATTCAT    2760
        ************************************************************

JG      GGGTATCAGCTCGCATCATACCTAAAACAGATGAATGCTAATCGAAATCCTTACTCTAGC    2820
YH      GGGTATCAGCTCGCATCATACCTAAAACAGATGAATGCTAATCGAAATCCTTACTCTAGC    2820
        ************************************************************

JG      ATGCCATTGGACCCACAGCGACTTCCAAAATCTTCTGCATCGGCTGTGCCAACCTATGTC    2880
YH      ATGCCATTGGACCCACAGCGACTTCCAAAATCTTCTGCATCGGCTGTGCCAACCTATGTC    2880
        ************************************************************

JG      GATTCTGTCATGCATGCTCGTAACCAGAACTTGCTTGCTTCATTGGGAGCTACTCCTTCA    2940
YH      GATTCTGTCATGCATGCTCGTAACCAGAACTTGCTTGCTTCATTGGGAGCTACTCCTTCA    2940
        ************************************************************

JG      CAGATCGCAGCAACATCCCGGATAGGTACTATGATGGCAGAAAGATCCTATTATGTCCCT    3000
YH      CAGATCGCAGCAACATCCCGGATAGGTACTATGATGGCAGAAAGATCCTATTATGTCCCT    3000
        ************************************************************

JG      TCCACTCTTGACGGGAATGAAAATGCTGGTTCATCAGCTTACTCAAAGAAGTACCACAGC    3060
YH      TCCACTCTTGACGGGAATGAAAATGCTGGTTCATCAGCTTACTCAAAGAAGTACCACAGC    3060
        ************************************************************
```

```
JG      TCACCAGACATATCTGCACTGATTGCTGCAAGCAGGAGTGCTCTGTTGAATGAATCAAAG    3120
YH      TCACCAGACATATCTGCACTGATTGCTGCAAGCAGGAGTGCTCTGTTGAATGAATCAAAG    3120
        ************************************************************

JG      TTGGGTGGTGGTACCATTGGATCCCAGTCGTACCTTAGCAGGCTTGCATCGGAAAGATCT    3180
YH      TTGGGTGGTGGTACCATTGGATCCCAGTCGTACCTTAGCAGGCTTGCATCGGAAAGATCT    3180
        ************************************************************

JG      CAGTATACAAACTCGGTGGCCAGGCCTGCAGCTCCCTTGGCGTTTGATGAGCTCTCTCCA    3240
YH      CAGTATACAAACTCGGTGGCCAGGCCTGCAGCTCCCTTGGCGTTTGATGAGCTCTCTCCA    3240
        ************************************************************

JG      CCTAAGCTCCCAGGGGATATCTTCTCAATGCAACAAAGCCCAAACCCAAGTGCAAGATCC    3300
YH      CCTAAGCTCCCAGGGGATATCTTCTCAATGCAACAAAGCCCAAACCCAAGTGCAAGATCC    3300
        ************************************************************

JG      CTTTGGGCTAAGCAACCTTTTGAGCAGCTGTTTGGTGTGTCGAGTGCGGAGCTCACTAAA    3360
YH      CTTTGGGCTAAGCAACCTTTTGAGCAGCTGTTTGGTGTGTCGAGTGCGGAGCTCACTAAA    3360
        ************************************************************

JG      AGCGAGTTCAACCCTGCAGGCAGATCGGGTGGCATGACCAAGGATGATTTCTCTTACAAG    3420
YH      AGCGAGTTCAACCCTGCAGGCAGATCGGGTGGCATGACCAAGGATGATTTCTCTTACAAG    3420
        ************************************************************

JG      GAGTCTGAGGCGAAGCTTCTTCAGTCTCTTAGATTCTGCATCTCGAAGCTCCTGAAGCTA    3480
YH      GAGTCTGAGGCGAAGCTTCTTCAGTCTCTTAGATTCTGCATCTCGAAGCTCCTGAAGCTA    3480
        ************************************************************

JG      GAAGGATCAGGGTGGCTGTTCAAGCAAAATGGTGGCAGCGACGAAGATCTGATTGATCAA    3540
YH      GAAGGATCAGGGTGGCTGTTCAAGCAAAATGGTGGCAGCGACGAAGATCTGATTGATCAA    3540
        ************************************************************

JG      GTTGCTGCGGTAGAGAAGCTATTGCAACAAGGAACCAGTGACAACCAACTGCTGCTTGGT    3600
YH      GTTGCTGCGGTAGAGAAGCTATTGCAACAAGGAACCAGTGACAACCAACTGCTGCTTGGT    3600
        ************************************************************

JG      GATACTCAGCAACCACCATGTGATAAGGCAGACATCCAGTACATGCGCGTACTGCCTAAC    3660
YH      GATACTCAGCAACCACCATGTGATAAGGCAGACATCCAGTACATGCGCGTACTGCCTAAC    3660
        ************************************************************

JG      TGCGGAGACGACTGCATCTGGCGCGCCTCCCTCGTTGTCAGCTTCGGTGTCTGGTGCATC    3720
YH      TGCGGAGACGACTGCATCTGGCGCGCCTCCCTCGTTGTCAGCTTCGGTGTCTGGTGCATC    3720
        ************************************************************

JG      CGCCGGGTGCTAGACCTGTCTCTGGTGGAAAGCAGGCCAGAACTTTGGGGCAAGTATACC    3780
YH      CGCCGGGTGCTAGACCTGTCTCTGGTGGAAAGCAGGCCAGAACTTTGGGGCAAGTATACC    3780
        ************************************************************

JG      TATGTTCTCAACCGTCTTCAGGGCATCCTGGATCCTGCATTCACCAAGCCTCGGAGTGCT    3840
YH      TATGTTCTCAACCGTCTTCAGGGCATCCTGGATCCTGCATTCACCAAGCCTCGGAGTGCT    3840
        ************************************************************

JG      CTCAGCGCGTGTGCGTGCCTTCACAGAGATATCCGGGTGCTCAACAGCCTGCGCCACAGT    3900
YH      CTCAGCGCGTGTGCGTGCCTTCACAGAGATATCCGGGTGCTCAACAGCCTGCGCCACAGT    3900
        ************************************************************

JG      AGCCTGGTAGCAACAAACTCCATTCCAAGGCAAATCCGAGGTTCCTTCACCACCGCATCT    3960
YH      AGCCTGGTAGCAACAAACTCCATTCCAAGGCAAATCCGAGGTTCCTTCACCACCGCATCT    3960
        ************************************************************

JG      GTGGTCCTGGAGATGATCAAGGATGTGGAGACCGCAGTCTCAGGGCGCAAGGGCAGGAGT    4020
YH      GTGGTCCTGGAGATGATCAAGGATGTGGAGACCGCAGTCTCAGGGCGCAAGGGCAGGAGT    4020
        ************************************************************

JG      GGCACCGCAGCTGGGGATGTCGCCTTCCCCAAGGGGAAGGAGAACCTGGCCTCCGTGCTG    4080
YH      GGCACCGCAGCTGGGGATGTCGCCTTCCCCAAGGGGAAGGAGAACCTGGCCTCCGTGCTG    4080
        ************************************************************

JG      AAGCGATACAAGCGGAGGCTCTCGAGCAAGGGACAACAATAATAAGGCATCTGGGCAGCG    4140
```

```
YH      AAGCGATACAAGCGGAGGCTCTCGAGCAAGGGACAACAATAATAAGGCATCTGGGCAGCG    4140
        ************************************************************

JG      TGATCCTGTCGCGTTTTAGGGGGACTTTGACCATTGTTCTTCAAGGATGGCAGCCAGCCA    4200
YH      TGATCCTGTCGCGTTTTAGGGGGACTTTGACCATTGTTCTTCAAGGATGGCAGCCAGCCA    4200
        ************************************************************

JG      TGGTGGCTTGCCCTCCCTGAGCCCTGGATTTTTTCGTTGCACAACGTTTGCAGGGACCTG    4260
YH      TGGTGGCTTGCCCTCCCTGAGCCCTGGATTTTTTCGTTGCACAACGTTTGCAGGGACCTG    4260
        ************************************************************

JG      AGGAATTGGCCAACACTTCTGGTCCCTTCCATCATATTTCGTTTTTTTTTGTTTCTTTCT    4320
YH      AGGAATTGGCCAACACTTCTGGTCCCTTCCATCATATTTCGTTTTTTTTTGTTTCTTTCT    4320
        ************************************************************

JG      TGTTTTTCTTTTTTGCATGTGATGTGTTGTATAATGGTAACTGTTCATGTGCCAGAAGAA    4380
YH      TGTTTTTCTTTTTTGCATGTGATGTGTTGTATAATGGTAACTGTTCATGTGCCAGAAGAA    4380
        ************************************************************

JG      CAACCACCAAAATGTACAACAGATGTAGTCAGCTGATGCACCATTGTAAAGTTTAGTCTC    4440
YH      CAACCACCAAAATGTACAACAGATGTAGTCAGCTGATGCACCATTGTAAAGTTTAGTCTC    4440
        ************************************************************

JG      TGCATTTTAACCTTTTTTTTGGGGGTCATTGACAAACTGAATGAATGCCCTGTGTAATCT    4500
YH      TGCATTTTAACCTTTTTTTTGGGGGTCATTGACAAACTGAATGAATGCCCTGTGTAATCT    4500
        ************************************************************

JG      CTCTTCAGAGAGGATGCCAAGACTGAGAAAAAGCTTTTGCCAGATTTCCAGATGCCTTGT    4560
YH      CTCTTCAGAGAGGATGCCAAGACTGAGAAAAAGCTTTTGCCAGATTTCCAGATGCCTTGT    4560
        ************************************************************

JG      GTTCAAAA        4568
YH      GTTCAAAA        4568
        ********
```
