# Supplementary material for: Comparison of leaf transcriptome in response to Rhizoctonia solani infection between resistant and susceptible rice cultivars
Source: BMC Genomics. 2020 Mar 19;21:245. doi: 10.1186/s12864-020-6645-6 (PMC7081601; doi:10.1186/s12864-020-6645-6)
Supplement: Supplementary file 9 — Additional file 9: Table S1. Primers for real-time quantitative PCR of selected DEGs. Table S2. Summary of RNA sequencing results. Table S3. FPKM values of selected DEGs. Data show mean ± standard error of FPKM values. * indicates significantly difference between infected and uninfected samples (P < 0.05). [file 12864_2020_6645_MOESM9_ESM.docx]

**Table S1. Primers for real-time quantitative PCR of selected DEGs.**

| Gene ID | Annotations | Sequence (5'to3') |
| --- | --- | --- |
| Os06g0666600 | glyceraldehyde-3-phosphate dehydrogenase, chloroplastic (GAPCP1) | TTGGTACGACAACGAGTGGG |
|  |  | CGGCAATGGTTTTGTTCGGT |
| Os03g0131200 | catalase-1 (CAT1) | CGTACTACTAGGCCTGCGTG |
|  |  | TAGCGGGCCACAAGAGAAAG |
| Os03g0418000 | chitinase 12 (CHI 12) | GGTCCCATCCAGCTCTCCTA |
|  |  | GGCGTCATCCAGAACCAGAA |
| Os07g0142900 | Uncharacterized oxidoreductase At1g06690, chloroplastic (OXD) | AGAGGAGTTCCACTTGCTGC |
|  |  | AGCTCATCACAAGCTGCCTT |
| Os05g0548900 | phosphomethylethanolamine N-methyltransferase (PEAMT) | AATGGCTGAAGGTAGGTGGC |
|  |  | AACCTTGGCTCCCGGTAATG |
| Os01g0382400 | pathogenesis-related protein PRB1-2 | CGCGCTGTGTGTTTGTGTTA |
|  |  | CGCATCGATCGTGGTTTTGT |
| Os07g0416900 | fatty acid desaturase DES2 (FAD2) | CTTCATCGCCATCTCGGACA |
|  |  | CAGGTACGTGACGACCACAA |
| LOC9267508 | WRKY22 | GCTGAAGAAAGTGGTGTGCG |
|  |  | GGCGAGCCCTTGATAGGTTT |
| Os08g0108100 | pectinesterase inhibitor 28-like (PEI) | ATCCTGTGTGGTGCTGAGTG |
|  |  | AGCTTCAGCACGGCAGTAAT |
| Os03g0826900 | uncharacterized LOC4334644 | GACGATCACCCGTACCAGTC |
|  |  | GTCTCGTTCAGGAATGCCCA |
| Os02g0678200 | SPX domain-containing membrane protein (SPX-MP) | ACCTCTCACTACTCCGTCCC |

|  |  | GGGACAGGTTCACTCCTTCG |
| --- | --- | --- |

**Table S2. Summary of RNA sequencing results.**

| Sample | Total Raw Reads (M) | Total Clean Reads (M) | Total Clean Bases (Gb) | Clean Reads Ratio (%) | Q20 (%) | Q30 (%) |
| --- | --- | --- | --- | --- | --- | --- |
| JG-0-1 | 65.17 | 61.47 | 6.15 | 94.32 | 97.22 | 89.59 |
| JG-0-2 | 67.68 | 62.91 | 6.29 | 92.95 | 96.91 | 88.55 |
| JG-0-3 | 65.17 | 60.97 | 6.10 | 93.56 | 97.12 | 88.88 |
| JG-1-1 | 67.67 | 62.86 | 6.29 | 92.89 | 97.00 | 88.57 |
| JG-1-2 | 65.17 | 61.13 | 6.11 | 93.81 | 97.23 | 89.43 |
| JG-1-3 | 65.17 | 61.02 | 6.10 | 93.64 | 97.19 | 89.21 |
| YH-0-1 | 65.17 | 61.15 | 6.12 | 93.83 | 97.27 | 89.58 |
| YH-0-2 | 65.17 | 60.95 | 6.09 | 93.52 | 97.22 | 89.38 |
| YH-0-3 | 67.67 | 62.78 | 6.28 | 92.76 | 97.08 | 88.84 |
| YH-1-1 | 65.17 | 61.29 | 6.13 | 94.05 | 97.31 | 89.46 |
| YH-1-2 | 67.68 | 62.74 | 6.27 | 92.70 | 96.95 | 88.50 |
| YH-1-3 | 67.67 | 63.05 | 6.31 | 93.17 | 97.16 | 89.24 |

**Table S3. FPKM values of selected DEGs.** Data show mean ± standard error of FPKM values. * indicates significantly difference between infected and uninfected samples (P<0.05).

| **DEGs** | **Description** | **JG-0** | **JG-1** | **YH-0** | **YH-1** |
| --- | --- | --- | --- | --- | --- |
| **ko00940** | **Phenylpropanoid biosynthesis** |  |  |  |  |
| 4.3.1.24 | phenylalanine ammonia-lyase (PAL) | 1931.71 ± 2771.97 | 10152.89 ± 7862.02 | 3263.49 ± 2968.98 | 6406.84 ± 5318.66 |
| 4.3.1.25 | phenylalanine/tyrosine ammonia-lyase (PTAL) | 1743.91 ± 2570.52 | 9463.38 ± 7403.28 | 3217.99 ± 2936.02 | 6390.38 ± 5307.63 |
| 1.14.1491 | CYP73A, trans-cinnamate 4-monooxygenase | 0.17 ± 0.16 | 0.50 ± 0.41 | 18.86 ± 6.04 | 50.59 ± 61.21 |
| 6.2.1.12 | 4-coumarate--CoA ligase (4CL) | 5.93 ± 5.64 | 14.75 ± 14.66 | 12.80 ± 4.28 | 28.09 ± 31.09 |
| 3.2.1.21 | beta-glucosidase | 132.78 ± 46.73 | 302.97 ± 64.04 | 75.76 ± 25.95 | 121.86 ± 107.59 |
| 1.1.1.195 | cinnamyl-alcohol dehydrogenase (CAD) | 23.48 ± 6.10 | 76.1 ± 43.47* | 9.43 ± 2.83 | 68.02 ± 85.61 |
| F5H | ferulate-5-hydroxylase (F5H) | 2.19 ± 1.55 | 5.41 ± 6.37 | 1.97 ± 0.45 | 8.09 ± 8.76 |
| 1.2.1.68 | redox factor 1 (REF1) | 2.50 ± 0.63 | 10.82 ± 5.97* | 2.31 ± 1.46 | 10.73 ± 13.82 |
| 1.14.11.61 | Feruloyl-CoA 6-hydroxylase (F6’H) | 10.28 ± 6.75 | 22.29 ± 8.69 | 0.96 ± 0.36 | 2.33 ± 3.05 |
| 23.1.133 | shikimate O-hydroxycinnamoyltransferase (HCT) | 2.62 ± 1.15 | 14.04 ± 8.00* | 6.12 ± 5.02 | 16.78 ± 25.96 |
| 1.11.1.7 | peroxidase (POD) | 3620.44 ± 275.62 | 2623.86 ± 322.06* | 3059.08 ± 930.04 | 1631.60 ± 696.62* |
| **ko04016** | **MAPK signaling pathway-plant** |  |  |  |  |
| FLS2 | LRR receptor-like serine/threonine-protein kinase FLS2 | 55.42 ± 14.12 | 43.45 ± 16.42* | 67.39 ± 19.22 | 37.86 ± 10.19* |
| MKS1 | MAP kinase substrate 1 | 6.76 ± 8.65 | 22.91 ± 29.21 | 0.03 ± 0.05 | 0.48 ± 0.46 |
| VIP1 | transcription factor VIP1 | 6.51 ± 0.78 | 20.63 ± 15.37 | 4.92 ± 1.54 | 8.02 ± 5.62 |
| WRKY22/29 | WRKY transcription factor 22 | 31.37 ± 11.53 | 15.17 ± 6.18* | 30.09 ± 8.97 | 12.27 ± 1.79* |
| PR1 | pathogenesis-related protein 1 | 11.17 ± 2.79 | 66.93 ± 28.68* | 67.52 ± 97.13 | 425.52 ± 510.22 |
| EIN3/EIL | ethylene-insensitive protein 3 | 2.40 ± 1.17 | 0.84 ± 0.08* | 2.02 ± 1.89 | 0.78 ± 0.42 |
| ERF1 | ethylene-responsive transcription factor 1 | 1.79 ± 1.04 | 13.59 ± 3.79* | 1.75 ± 0.46 | 20.26 ± 18.7 |
| CHIB | endochitinase B | 493.14 ± 168.30 | 1194.13 ± 442.33* | 191.20 ± 14.02 | 379.99 ± 428.85 |
| VSP2 | vegetative storage protein 2 | 14.63 ± 22.91 | 163.08 ± 147.09 | 11.16 ± 10.15 | 12.35 ± 13.64 |
| PYR/PYL | abscisic acid receptor PYR/PYL family | 1.72 ± 2.98 | 0.23 ± 0.27 | 17.77 ± 22.97 | 1.37 ± 0.29 |
| MAP3K17/18 | mitogen-activated protein kinase kinase kinase 17/18 | 7.30 ± 8.98 | 3.15 ± 2.54 | 167.79 ± 136.44 | 70.55 ± 65.94 |
| WRKY33 | WRKY transcription factor 33 | 38.31 ± 42.12 | 104.05 ± 123.88 | 783.72 ± 177.71 | 382.85 ± 178.47* |
| FRK1 | senescence-induced receptor | 10.75 ± 9.25 | 4.25 ± 5.84 | 2.82 ± 0.94 | 6.55 ± 2.76* |
| EIN2 | ethylene-insensitive protein 2 | 14.04 ± 4.53 | 5.34 ± 3.38* | 32.00 ± 3.02 | 41.33 ± 4.89* |
| MYC2 | transcription factor MYC2 | 0.18 ± 0.07 | 1.53 ± 1.42 | 232.45 ± 189.03 | 66.08 ± 77.79 |
| PP2C | protein phosphatase 2C | 8.92 ± 4.57 | 2.61 ± 1.58* | 0.14 ± 0.09 | 2.03 ± 3.41 |
| MEKK1 | mitogen-activated protein kinase kinase kinase 1 | 15.71 ± 6.32 | 5.47 ± 2.93* | 0.86 ± 1.16 | 3.42 ± 4.41 |
| CAT1 | catalase | 273.83 ± 191.56 | 42.03 ± 23.55* | 89.72 ± 71.09 | 37.88 ± 31.09 |
| CAM4 | calmodulin | 268.82 ± 39.69 | 148.10 ± 52.81* | 0.28 ± 0.08 | 0.96 ± 1.62 |
| ER/ERLs | LRR receptor-like serine/threonine-protein kinase ERECTA | 32.37 ± 23.6 | 42.29 ± 24.53 | 30.11 ± 23.07 | 22.83 ± 16.05 |
| SPCH | transcription factor SPEECHLESS | 6.02 ± 4.78 | 4.13 ± 1.53 | 4.42 ± 6.26 | 0.68 ± 0.38 |
| MKK4/5 | mitogen-activated protein kinase kinase 4/5 | 1.35 ± 0.76 | 1.25 ± 0.91 |  |  |
| OXI1 | serine/threonine-protein kinase OXI1 | 0.12 ± 0.12 | 0.50 ± 0.39 |  |  |
| ETR/ERS | ethylene receptor | 4.12 ± 2.98 | 1.90 ± 1.42 |  |  |
| EBF1/2 | EIN3-binding F-box protein | 171.41 ± 73.50 | 82.59 ± 32.80 |  |  |
| RBOHD | respiratory burst oxidase | 2.53 ± 2.71 | 4.71 ± 4.23 |  |  |
| ANP1 | mitogen-activated protein kinase kinase kinase ANP1 |  |  | 0.72 ± 0.73 | 0.29 ± 0.25 |
| ACS6 | 1-aminocyclopropane-1-carboxylate synthase 6 |  |  | 3.44 ± 1.28 | 8.33 ± 10.72 |
| SNRK2 | serine/threonine-protein kinase SRK2 |  |  | 167.79 ± 136.44 | 70.55 ± 65.94 |
| **ko04626** | **Plant-pathogen interaction** |  |  |  |  |
| CDPK | calcium-dependent protein kinase | 102.41 ± 100.87 | 90.67 ± 91.79 |  |  |
| Rboh | respiratory burst oxidase | 2.53 ± 2.71 | 4.71 ± 4.23 |  |  |
| CNGCs | cyclic nucleotide gated channel, plant | 21.17 ± 16.19 | 36.13 ± 27.06 |  |  |
| CAM/CML | calmodulin | 274.77 ± 41.8 | 158.82 ± 56.69* |  |  |
| FLS2 | LRR receptor-like serine/threonine-protein kinase FLS2 | 55.42 ± 14.12 | 59.17 ± 14.62 |  |  |
| MEKK1 | mitogen-activated protein kinase kinase kinase 1 | 15.71 ± 6.32 | 5.47 ± 2.93* |  |  |
| MKK4/5 | mitogen-activated protein kinase kinase 4/5 | 1.35 ± 0.76 | 1.25 ± 0.91 |  |  |
| WRKY25/33 | WRKY transcription factor 25 | 38.31 ± 42.12 | 104.05 ± 123.88 |  |  |
| WRKY22/29 | WRKY transcription factor 22 | 31.37 ± 11.53 | 15.17 ± 6.18* |  |  |
| WRKY29 | WRKY transcription factor 29 | 0.51 ± 0.88 | 0.08 ± 0.08 |  |  |
| FRK1 | senescence-induced receptor-like serine/threonine-protein kinase | 10.75 ± 9.25 | 4.25 ± 5.84 |  |  |
| PR1 | pathogenesis-related protein 1 | 11.17 ± 2.79 | 66.93 ± 28.68* |  |  |
| EFR | LRR receptor-like serine/threonine-protein kinase EFR | 4.52 ± 3.62 | 5.91 ± 5.5 |  |  |
| Pti6 | pathogenesis-related genes transcriptional activator PTI6 | 0.41 ± 0.11 | 0.9 ± 0.57 |  |  |
| Pti1 | pto-interacting protein 1 | 0.24 ± 0.42 | 1.42 ± 1.64 |  |  |
| RIN4 | RPM1-interacting protein 4 | 1.05 ± 1.2 | 2.37 ± 1.91 |  |  |
| RPM1 | disease resistance protein RPM1 | 49.53 ± 20.64 | 49.73 ± 16.98 |  |  |
| RPS2 | disease resistance protein RPS2 | 7.28 ± 2.96 | 17.61 ± 16.35 |  |  |
| PBS1 | serine/threonine-protein kinase PBS1 | 1.31 ± 1.16 | 2.78 ± 1.51 |  |  |
| HSP90 | heat shock protein 90kDa beta | 76.03 ± 104.58 | 33.35 ± 21.16 |  |  |
| EIX1/2 | EIX receptor 1/2 | 2.68 ± 1.58 | 1.83 ± 1.21 |  |  |
| XA21 | receptor kinase-like protein | 26.71 ± 16.47 | 19.94 ± 16.62 |  |  |
| CEBIP | chitin elicitor-binding protein | 0.11 ± 0.17 | 0.67 ± 0.62 |  |  |
| CERK1 | chitin elicitor receptor kinase 1 | 0.66 ± 0.24 | 1.55 ± 0.42 |  |  |
| RRS1-R | probable WRKY transcription factor 52 | 10.60 ± 6.15 | 25.45 ± 29.08 |  |  |
| WRKY1/2 | WRKY transcription factor 1 | 30.96 ± 33.22 | 83.03 ± 95.42 |  |  |
